# Cation Distribution and Anion Transport in the La_3_Ga_5–*x*_Ge_1+*x*_O_14+0.5*x*_ Langasite Structure

**DOI:** 10.1021/jacs.4c02324

**Published:** 2024-05-08

**Authors:** Lucia Corti, Ivan Hung, Amrit Venkatesh, Zhehong Gan, John B. Claridge, Matthew J. Rosseinsky, Frédéric Blanc

**Affiliations:** † Department of Chemistry, University of Liverpool, Liverpool L69 7ZD, U.K.; ‡ Leverhulme Research Centre for Functional Materials Design, Materials Innovation Factory, University of Liverpool, Liverpool L69 7ZD, U.K.; § National High Magnetic Field Laboratory, Florida State University, Tallahassee, Florida 32310, United States; ∥ Stephenson Institute for Renewable Energy, University of Liverpool, Liverpool L69 7ZF, U.K.

## Abstract

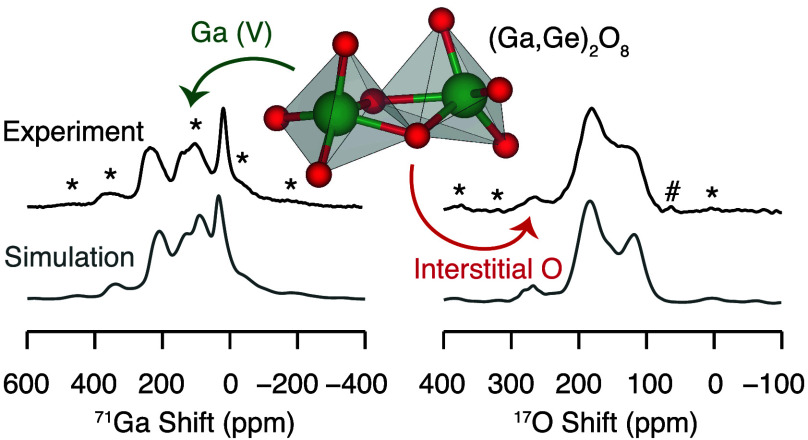

Exploration of compositional disorder using conventional diffraction-based techniques is challenging for systems containing isoelectronic ions possessing similar coherent neutron scattering lengths. Here, we show that a multinuclear solid-state Nuclear Magnetic Resonance (NMR) approach provides compelling insight into the Ga^3+^/Ge^4+^ cation distribution and oxygen anion transport in a family of solid electrolytes with langasite structure and La_3_Ga_5–*x*_Ge_1+*x*_O_14+0.5*x*_ composition. Ultrahigh field ^71^Ga Magic Angle Spinning (MAS) NMR experiments acquired at 35.2 T offer striking resolution enhancement, thereby enabling clear detection of Ga sites in different coordination environments. Three-connected GaO_4_, four-connected GaO_4_ and GaO_6_ polyhedra are probed for the parent La_3_Ga_5_GeO_14_ structure, while one additional spectral feature corresponding to the key (Ga,Ge)_2_O_8_ structural unit which forms to accommodate the interstitial oxide ions is detected for the Ge^4+^-doped La_3_Ga_3.5_Ge_2.5_O_14.75_ phase. The complex spectral line shapes observed in the MAS NMR spectra are reproduced very accurately by the NMR parameters computed for a symmetry-adapted configurational ensemble that comprehensively models site disorder. This approach further reveals a Ga^3+^/Ge^4+^ distribution across all Ga/Ge sites that is controlled by a kinetically governed cation diffusion process. Variable temperature ^17^O MAS NMR experiments up to 700 °C importantly indicate that the presence of interstitial oxide ions triggers chemical exchange between all oxygen sites, thereby enabling atomic-scale understanding of the anion diffusion mechanism underpinning the transport properties of these materials.

## 1 Introduction

Solid Oxide Fuel Cells (SOFCs) are promising all-solid-state power generation devices enabling the electrochemical conversion of chemical energy into electric energy and represent one of the key technologies which are being considered to address the rapidly increasing global energy demand. One of the main advantages of SOFCs compared to other types of fuel cells is the ability of this device to operate on a wide range of fuels, including but not limited to hydrogen.^1^ Nevertheless, the further development of SOFCs relies on the reduction of their operating temperature to intermediate (650 °C–800 °C) or even lower (below 650 °C) ranges,^1^ and research effort has been undertaken to identify suitable solid electrolytes that exhibit elevated oxide ion conductivity at these temperatures.^2^

The presence of chemical defects in the lattice is associated with increased ionic conductivity, and oxide materials are commonly doped with aliovalent cations to form oxygen vacancies or interstitials that lead to enhanced transport properties. While the most widely used solid oxide electrolytes adopt fluorite^3,4^ or perovskite^5^ structure with oxygen vacancies driving the ionic diffusion, there has been a growing interest in the development of solid electrolytes with a flexible framework that are able to accommodate interstitial oxygens, and this has led to the discovery of oxide ion transport materials with melilite^6^ and langasite^7^ structures among others.^8−10^

**Figure 1:**
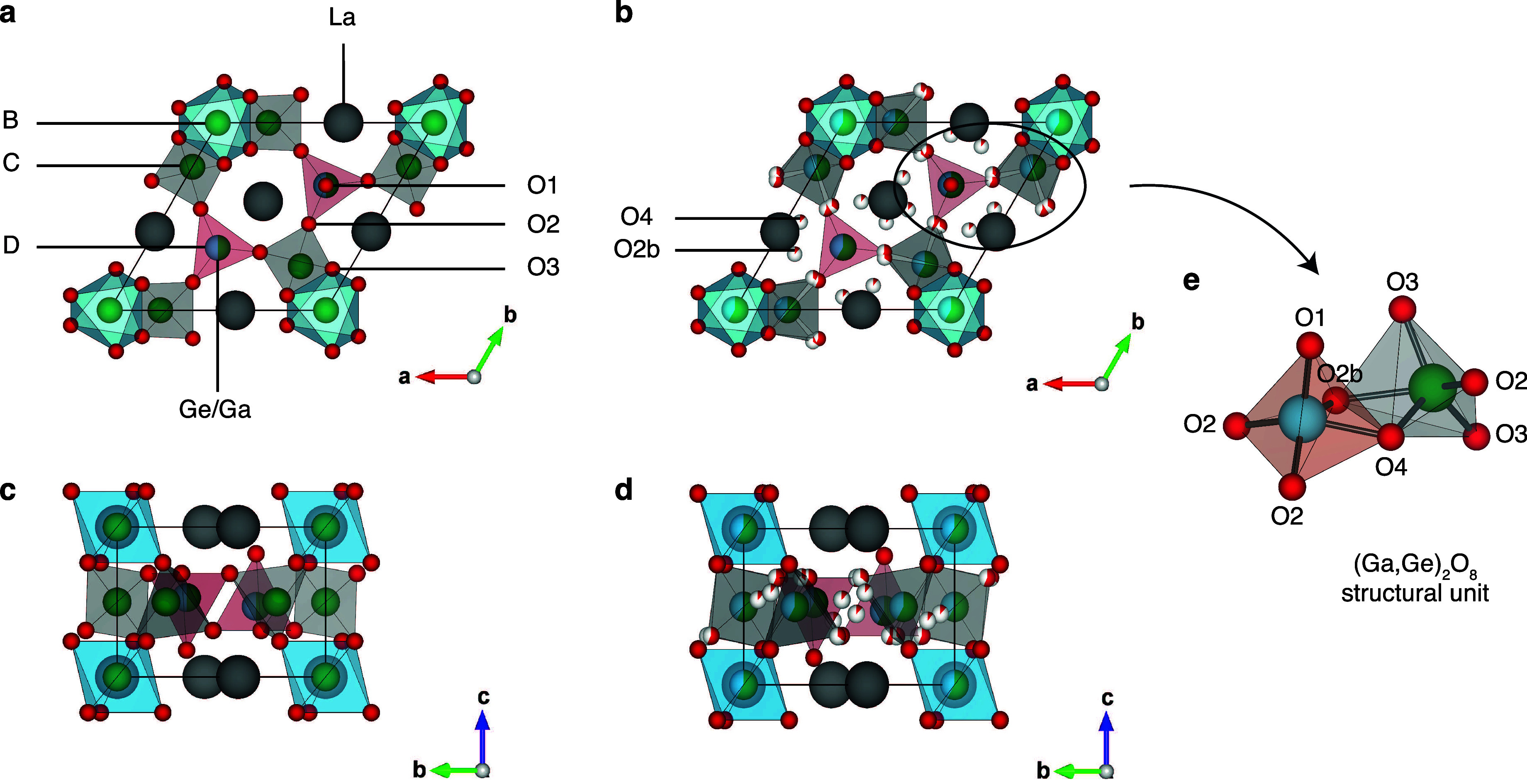
Structures viewed along the (a, b) *c*-axis and the (c, d) *a*-axis of (a, c) La_3_Ga_5_GeO_14_ and (b, d) La_3_Ga_3.5_Ge_2.5_O_14.75_.^7^ O, Ga, Ge and La atoms are shown in red, green, blue and gray. Three-connected DO_4_ tetrahedra (red) with one nonbridging oxide ion O1 are connected to three four-connected CO_4_ tetrahedra (gray) via O2 ions, and BO_6_ octahedra (blue) bridge four-connected CO_4_ tetrahedra belonging to adjacent layers via O3 ions. In La_3_Ga_5_GeO_14_, B and C sites are fully occupied by Ga^3+^ cations, while the D site exhibits Ga^3+^/Ge^4+^ mixed site occupancy, as reported in refs (11−13). In La_3_Ga_3.5_Ge_2.5_O_14.75_, Ga^3+^/Ge^4+^ cations are distributed across the B, C, and D sites.^7^ (e) An example of (Ga,Ge)_2_O_8_ structural unit which forms upon Ge^4+^ doping. Ga^3+^/Ge^4+^ cations are randomly distributed within the (Ga,Ge)_2_O_8_ unit.

The La_3_Ga_5_GeO_14_ langasite structure (general formula A_3_BC_3_D_2_O_14_) consists of layers of three-connected DO_4_ tetrahedra distinguished by the presence of one nonbridging oxide ion and four-connected CO_4_ tetrahedra containing four bridging oxide ions (Figure 1a and 1c). These layers are connected to form a three-dimensional framework by BO_6_ octahedra which bridge four-connected CO_4_ tetrahedra belonging to adjacent layers. The void space between the tetrahedral layers is occupied by eight-coordinate La^3+^ cations (A sites) located in hexagonal channels formed by the edges of one BO_6_ octahedron, three four-connected CO_4_ tetrahedra and two three-connected DO_4_ tetrahedra. It has been reported that B and C sites in La_3_Ga_5_GeO_14_ are fully occupied by Ga^3+^, while a 50/50 mixture of Ga^3+^/Ge^4+^ occupies the D site.^11−13^ Contrasting results were obtained in further work on a multicell model of La_3_Ga_5_GeO_14_, wherein it was concluded that Ge^4+^ cations partially occupy both B and D sites.^14^ Interstitial oxide ions introduced in the lattice upon Ge^4+^-doping to form La_3_Ga_5–*x*_Ge_1+*x*_O_14+0.5*x*_ are accommodated in a (Ga,Ge)_2_O_8_ structural unit consisting of a pair of edge-sharing five-coordinate Ga/Ge square pyramidal sites connected via one interstitial oxide ion O4 and one framework oxide ion which is displaced from its original O2 position to the O2b site (Figure 1b, 1d, and 1e).^7^ It has been reported that B, C, and D sites in La_3_Ga_5–*x*_Ge_1+*x*_O_14+0.5*x*_ with *x* > 0 exhibit Ga^3+^/Ge^4+^ mixed site occupancies.^7^

Importantly, the maximum amount of excess oxygen that can be incorporated in the La_3_Ga_5–*x*_Ge_1+*x*_O_14+0.5*x*_ langasite structure (i.e., up to 5.4 mol % in La_3_Ga_3.5_Ge_2.5_O_14.75_ with respect to the amount of oxygen in La_3_Ga_5_GeO_14_) exceeds the concentration of interstitial defects in the related tetragonal La_1+*y*_Sr_1–*y*_Ga_3_O_7+0.5*y*_ melilite phase with highest concentration of dopant (i.e., up to 3.8 mol % in La_1.54_Sr_0.46_Ga_3_O_7.27_ versus LaSrGa_3_O_7_ while preserving tetragonal structure). Nevertheless, a comparison of the transport properties in La_3_Ga_5–*x*_Ge_1+*x*_O_14+0.5*x*_ and La_1+*y*_Sr_1–*y*_Ga_3_O_7+0.5*y*_ at 500 °C shows that the oxide ion conductivity is 2 orders of magnitude higher in La_1.54_Sr_0.46_Ga_3_O_7.27_ than in the most highly conductive langasite phase.^7^ Furthermore, the ionic conductivity as a function of excess oxygen concentration increases less significantly in the langasites than in the melilites and is observed to decrease in La_3_Ga_5–*x*_Ge_1+*x*_O_14+0.5*x*_ with *x* > 0.45.^7^ These observations suggest that the incorporation of interstitial oxide ions in La_3_Ga_5–*x*_Ge_1+*x*_O_14+0.5*x*_ leads to a substantial structural rearrangement, and the formation of (Ga,Ge)_2_O_8_ units effectively traps the interstitial ions, thereby limiting the enhancement in ionic conductivity occurring upon Ge^4+^ doping.^7^

Although the ionic conductivity in La_3_Ga_5–*x*_Ge_1+*x*_O_14+0.5*x*_ is lower than that measured for state-of-the-art solid oxide electrolytes, the langasite family offers great flexibility with regards to the range of cations that can occupy the lattice sites.^15^ The distribution of cations among the distinct polyhedra can be tuned to reduce the structural rearrangements which occur upon Ge^4+^ doping and limit the excessive stabilization of the interstitial defects in the (Ga,Ge)_2_O_8_ units. This motivates further examination of the compositional disorder in the site-disordered langasite family and highlights the need to investigate Ga^3+^/Ge^4+^ cation distribution in the Ge^4+^-doped phase to identify possible relations between the local structure and the oxide ion conduction mechanism. As exemplified by previous work, it is very challenging to resolve the Ga^3+^/Ge^4+^ cation distribution across the B, C, and D sites in langasite structures using conventional X-ray and neutron diffraction methods due to the absence of X-ray scattering contrast for this isoelectronic pair and the similar coherent neutron scattering lengths of Ga (7.3 fm) and Ge (8.2 fm).^7,11−14,16,17^ Solid-state Nuclear Magnetic Resonance (NMR) spectroscopy is element-specific, thus offering an alternative approach to investigate compositional disorder in La_3_Ga_5–*x*_Ge_1+*x*_O_14+0.5*x*_.

Solid-state NMR spectroscopy is highly sensitive to changes in the local environment around the nuclei being probed, making this technique ideal to access the local structure in La_3_Ga_5–*x*_Ge_1+*x*_O_14+0.5*x*_. ^17^O (spin quantum number ) Magic Angle Spinning (MAS) NMR spectroscopy represents a crucial technique to unravel the local structure around the oxygen sites, the key element in oxide ion conductors, and ^17^O Variable Temperature (VT) MAS NMR experiments have proven to be extremely powerful to gain insight into the local dynamics across a wide range of time scales and identify the oxide ion diffusion mechanism in solid electrolytes.^18−24^ Importantly, oxide ion conductors can be readily ^17^O enriched via a post synthetic exchange procedure based on high temperature annealing with ^17^O enriched O_2_ gas to overcome the limitations of the low natural abundance (0.037%) of the only NMR active isotope of oxygen, ^17^O.^25^^71^Ga (spin quantum number ) MAS NMR spectroscopy is well suited for structural elucidation by virtue of the established relation between the ^71^Ga isotropic chemical shift and the Ga coordination environment^26−28^ but requires high external magnetic field strengths and rapid sample spinning rates owing to relatively large nuclear electric quadrupole moment of ^71^Ga (NMR properties listed in Table S1 in the Supporting Information). Previous ^71^Ga NMR work on La_3_Ga_5–*x*_Ge_1+*x*_O_14+0.5*x*_ at 20 T and under MAS rates ν_r_ = 65 kHz revealed the presence of GaB octahedra and GaD tetrahedra in the parent phase, while one additional ^71^Ga signal tentatively assigned to five-coordinate GaD centers was detected upon Ge^4+^-doping.^7^ Nevertheless, the relative area of the signals in the ^71^Ga MAS NMR spectrum of La_3_Ga_5_GeO_14_ diverges from the 1:1 ratio expected based on the percentage of GaB and GaD sites in the average unit cell, and GaC polyhedra were not detected owing to the large quadrupolar coupling constant *C*_Q_ predicted for this site.^7,11^^139^La (spin quantum number ) is another NMR-active nucleus suitable to examine structural details,^26^ although ^139^La NMR spectroscopy is less frequently exploited due to the typically large ^139^La quadrupolar coupling constants that lead to extremely broad line shapes (Table S1).

The notoriously nontrivial interpretation of solid-state NMR spectra has fuelled growing interest in the computational prediction of the NMR parameters to aid spectral assignment of complex line shapes, and the Gauge Including Projector Augmented Waves (GIPAW)-Density Functional Theory (DFT) method is now commonly employed for periodic solids.^29−31^ The computational prediction of NMR parameters for site-disordered solids such as La_3_Ga_5–*x*_Ge_1+*x*_O_14+0.5*x*_ is challenged by the presence of fractional site occupancies in the average unit cell that are not effectively modeled in a single configuration. Such systems require computations to be carried out for a configurational ensemble, and the Site Occupancy Disorder (SOD) method,^32^ recently introduced to the field of NMR,^33^ enables the identification of all symmetrically inequivalent configurations for a given average unit cell.

Here, we explore the local structure and coordination environments of the ions in the undoped La_3_Ga_5_GeO_14_ and Ge^4+^-doped La_3_Ga_3.5_Ge_2.5_O_14.75_ langasites and tackle the compositional disorder of the Ga^3+^ and Ge^4+^ cations using solid-state NMR spectroscopy, thereby addressing the debated results obtained with diffraction-based methodologies. The inherent resolution limitations of half-integer quadrupolar nuclear spins such as ^71^Ga and ^139^La are overcome by performing the NMR experiments at ultrahigh magnetic fields with the Series Connected Hybrid (SCH) magnet operating at 35.2 T, thereby enabling the acquisition of highly resolved NMR spectra.^34^ The Ga^3+^/Ge^4+^ cation distribution is subsequently captured by comparing the experimental NMR data with the NMR spectra simulated for an ensemble of configurations which effectively models the possible distributions of the ions in the average unit cell. The results are exploited to interpret the evolution of the ^17^O MAS NMR spectra as a function of temperature up to 700 °C, establishing that the oxide ion diffusion involves all oxide ions and is mediated by the concerted rotation of the (Ga,Ge)O_*n*_ units.

## 2 Experimental Section

### 2.1 Materials Synthesis

La_3_Ga_5_GeO_14_ was synthesized using a standard procedure based on annealing at 1300 °C of a mixture of the binary oxide starting materials (La_2_O_3_, Ga_2_O_3_, and GeO_2_), and Ge^4+^-doped La_3_Ga_3.5_Ge_2.5_O_14.75_ was prepared using a sol–gel method which expands the chemical space and enables the incorporation of large concentrations of dopant (*x* ≥ 0.30) while preventing the formation of secondary phases, as described in detail elsewhere.^7^ To enable the acquisition of ^17^O MAS NMR experiments, the samples were ^17^O enriched using a standard method based on high-temperature, postsynthetic exchange with ^17^O_2_ gas.^25^ In particular, the samples were heated at 750 °C for 24 h in an atmosphere of 60% ^17^O enriched O_2_ gas (Isotec) using heating and cooling rates of 5 K min^–1^. The ^17^O level is expected to be ∼9% in La_3_Ga_5_GeO_14_ and ∼8% in La_3_Ga_3.5_Ge_2.5_O_14.75_ based on mass balance analysis between the langasite sample and the ^17^O enriched O_2_ gas used in the enrichment procedure. This assumes an equal mole fraction of the oxygen isotopes in the ^17^O enriched sample and in the ^17^O enriched atmosphere at the end of the labeling process.

### 2.2 Solid-State NMR Experiments

#### 2.2.1 ^71^Ga MAS NMR Experiments

^71^Ga MAS NMR experiments at 23.5 T were performed on a Bruker Avance Neo NMR spectrometer equipped with a double resonance 1.3 mm HX MAS probe tuned to X = ^71^Ga at a Larmor frequency ν_0_ = 305.11 MHz. One-dimensional spectra of La_3_Ga_5_GeO_14_ and La_3_Ga_3.5_Ge_2.5_O_14.75_ were acquired under MAS rates ν_r_ of 60 kHz with the rotor-synchronized Hahn echo pulse sequence, using Central Transition (CT)-selective pulses at a radio frequency (rf) field amplitude of 20 kHz and recycle delays of 2 s. ^71^Ga MAS NMR spectra at 23.5 T are reported relative to the ^71^Ga signal of a 1 M solution of Ga(NO_3_)_3_ in H_2_O at 0 ppm, also used to measure nutation frequencies.

Ultrahigh field ^71^Ga MAS NMR experiments were performed on the 36 T SCH magnet available at the National High Magnetic Field Laboratory (NHMFL) NHMFL in Tallahassee (Florida, USA) operating at 35.2 T.^34^ A Bruker Avance Neo console and a solid-state 1.3 mm HXY MAS NMR probe tuned to ^71^Ga at ν_0_ = 457.48 MHz were used to acquire the data, and samples were spun at ν_r_ = 60 kHz. One-dimensional ^71^Ga MAS NMR spectra were acquired with the rotor-synchronized Quadrupolar Carr–Purcell–Meiboom–Gill (QCPMG) pulse sequence^35−38^ combined with an initial Wideband Uniform Rate Smooth Truncation (WURST) shaped pulse^39^ for signal enhancement. The duration of the excitation and refocusing pulses was set to experimentally optimized values, respectively 1.25 and 2.5 μs for La_3_Ga_5_GeO_14_ and 1.5 and 3 μs for La_3_Ga_3.5_Ge_2.5_O_14.75_. The 1 ms WURST pulse was placed at an experimentally optimized frequency offset of 600 kHz, and the power of the frequency sweep was set to approximately 30 kHz. The envelope of the QCPMG spikelet pattern was obtained via Fourier transform of the coadded echoes. Truncating the QCPMG echo train did not lead do changes in the relative area of the signals, thereby revealing that the different Ga sites exhibit similar transverse relaxation time constants *T*_2_^′^ and confirming that the QCPMG spectra are quantitative.

A two-dimensional ^71^Ga spectrum of La_3_Ga_5_GeO_14_ was recorded with the Quadrupolar Magic-Angle Turning (QMAT) pulse sequence in combination with an initial WURST pulse and QCPMG acquisition mode for signal enhancement.^35−37,39,40^ The QMAT spectrum was recorded using CT-selective π/2 and π pulses of length equal to 1.25 and 2.5 μs, respectively. A total of 16 *t*_1_ increments were recorded, and the experimental conditions of the initial WURST pulse were kept the same as those in the corresponding one-dimensional spectrum. All ^71^Ga MAS NMR spectra recorded at 35.2 T were obtained with recycle delays suitable to obtain quantitative spectra (i.e., 2 s for La_3_Ga_5_GeO_14_ and 0.4 s for La_3_Ga_3.5_Ge_2.5_O_14.75_). NMR experiments at 35.2 T were externally calibrated to the ^1^H chemical shift of alanine at 1.46 ppm (indirectly referenced to tetramethylsilane at 0 ppm) using the IUPAC frequency ratios.^41^

#### 2.2.2 ^73^Ge NMR Experiments

^73^Ge NMR experiments were performed on a 20 T Bruker Neo Avance spectrometer equipped with a low-gamma 4 mm HX probe tuned to X = ^73^Ge at ν_0_ = 29.66 MHz. One-dimensional NMR spectra were acquired under static conditions using the WURST-QCPMG and Double Frequency Sweeps (DFS) DFS spin echo pulse sequences,^42−44^ and the experimental parameters were varied in an attempt to detect signal. The unfavorable NMR properties of ^73^Ge (see Table S1) precluded the observation of ^73^Ge resonances.

#### 2.2.3 ^17^O MAS NMR Experiments

Room-temperature ^17^O MAS NMR spectra at 20 T and under a MAS rate ν_r_ = 22 kHz were recorded using the experimental settings already detailed in previous work.^7^

^17^O VT MAS NMR experiments were performed on a 20 T Bruker Neo Avance spectrometer equipped with a 7 mm laser-heated single resonance X MAS probe^45^ tuned to X = ^17^O at a Larmor frequency ν_0_ = 115.28 MHz and under ν_r_ = 4 kHz. ^17^O MAS NMR experiments in the 19 °C–300 °C temperature range were additionally performed using a 4 mm high temperature double resonance HX MAS probe spinning at ν_r_ = 10 kHz for La_3_Ga_5_Ge^17^O_14_ and ν_r_ = 12.5 kHz for La_3_Ga_3.5_Ge_2.5_^17^O_14.75_ owing to the enhanced spectral resolution attainable with this probe. Unless otherwise specified, ^17^O VT NMR spectra were recorded with the pulse-acquire sequence using experimentally optimized 30° flip angle pulses at a rf field amplitude of either 20 kHz (7 mm probe) or 42 kHz (4 mm probe) and suitable recycle delays to obtain quantitative data. ^17^O MAS NMR spectra of La_3_Ga_5_Ge^17^O_14_ above 300 °C were acquired with experimentally optimized 90° flip angle pulses and recycle delays of approximately 1.3 times the spin–lattice relaxation time constant in the laboratory frame (*T*_1_) owing to the long *T*_1_ values determined for La_3_Ga_5_Ge^17^O_14_ and the need for an increased number of transients to obtain a satisfactory signal-to-noise ratio when using the laser-heated 7 mm probe as opposed to the 4 mm probe.

^17^O *T*_1_ values were determined from saturation recovery experiments performed with a saturation block consisting of a train of 90° flip angle pulses (100 for La_3_Ga_5_GeO_14_ and from 100 at room temperature to 10 at 700 °C for La_3_Ga_3.5_Ge_2.5_O_14.75_) with an rf field amplitude of 20 kHz separated by short, rotor-asynchronized (where applicable) time intervals δ (1.125 ms for La_3_Ga_5_GeO_14_ and from 0.875 ms at room temperature to 60 μs at 700 °C for La_3_Ga_3.5_Ge_2.5_O_14.75_) to ensure complete saturation of the spin system at each temperature and considering the probe safety.^46^ Suitable delays τ (e.g., at room temperature from 1 ms to 110 s for La_3_Ga_5_GeO_14_ and from 0.6 ms to 22 s for La_3_Ga_3.5_Ge_2.5_O_14.75_) were chosen at each temperature to fully capture the magnetization build-up. This build-up as a function of τ was fitted to the stretch exponential function shown in eq 1 to account for (i) the presence of overlapping signals which results in a distribution of *T*_1_ relaxation time constants and (ii) the temperature gradient across the sample
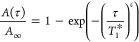
1where *A*(τ) and *A*_∞_ are the normalized area of the ^17^O overlapping signals respectively at delay τ and infinity, *T*_1_^*^ is the characteristic time constant, and *c* is the stretch exponent. *c* was constrained to the 0–1 range and was observed to take values between 0.472 and 0.994. Equation 2 enabled the determination of the mean *T*_1_ value from *T*_1_^*^ and *c*
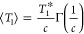
2where Γ is the gamma function. Since the τ values were not equally spaced, weights ω yielded from kernel density estimation were included in the fitting procedure as in eq 3
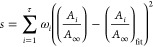
3where *s* represents the sum of the squared error which was minimized in the fit.

Temperature calibrations were performed using standard procedures based on the detection of the ^207^Pb chemical shift thermometer of Pb(NO_3_)_2_^47^ for the 4 mm high temperature HX MAS probe and the ^79^Br chemical shift thermometer of KBr^48^ for the 7 mm laser-heated X MAS probe. Variations in temperature across the rotor of up to ∼50 °C at 700 °C for the 7 mm probe and ∼7 °C at 280 °C for the 4 mm probe were detected from the corresponding temperature calibrations. All ^17^O experiments were acquired on ^17^O enriched samples and are referenced to the ^17^O signal of H_2_O at 0 ppm, also used to measure nutation frequencies.

#### 2.2.4 ^139^La NMR and MAS NMR Experiments

^139^La NMR experiments were performed at 35.2 T using the SCH magnet available at the NHMFL. A 1.3 mm HXY MAS NMR probe tuned to X = ^139^La at ν_0_ = 211.95 MHz was used throughout. All ^139^La NMR spectra were acquired using recycle delays of 0.5 s for La_3_Ga_5_GeO_14_ and 70 ms for La_3_Ga_3.5_Ge_2.5_O_14.75_. One-dimensional NMR spectra were recorded with the QCPMG pulse sequence^35−37^ both under static conditions and spinning the samples at ν_r_ = 60 kHz. Excitation and refocusing pulses of duration equal to 1.5 μs were used. QCPMG spectra recorded under MAS conditions were rotor-synchronized. Two-dimensional ^139^La QMAT spectra were recorded while spinning the samples at a MAS rate of 60 kHz and using the QCPMG acquisition mode for signal enhancement. CT-selective π/2 and π pulses of 1 and 2 μs in duration were used, and 16 *t*_1_ increments were recorded. ^139^La spectra were externally calibrated to the ^1^H chemical shift of alanine at 1.46 ppm (indirectly referenced to tetramethylsilane at 0 ppm) using the IUPAC frequency ratios.^41^

### 2.3 Computations

The complete set of symmetrically inequivalent configurations (i.e., not interconvertible via isometric transformations) from a La_3_Ga_5_GeO_14_ unit cell and a La_3_Ga_4_Ge_2_O_14.5_ 1 × 1 × 2 supercell (on the basis of the cell parameters of the La_3_Ga_4_Ge_2_O_14.5_ average unit cell obtained from diffraction measurements)^7^ was generated using the SOD method.^32^ A total of three symmetrically inequivalent configurations was obtained for La_3_Ga_5_GeO_14_ assuming Ga^3+^/Ge^4+^ mixed site occupancies for the three-connected DO_4_ tetrahedra, four-connected CO_4_ tetrahedra, and BO_6_ octahedra. 495 symmetrically inequivalent configurations were generated for La_3_Ga_4_Ge_2_O_14.5_ taking into account additional mixed site occupancy of the Ga^3+^/Ge^4+^ sites in the (Ga,Ge)_2_O_8_ structural unit and the partial site occupancy of the O2, O2b, and O4 sites depicted in Figure 1b and 1d, while forcing the oxide ion originally located in the O2 site of the undoped phase to occupy the O2b site in the presence of an interstitial oxide ion O4 nearby. The La_3_Ga_4_Ge_2_O_14.5_ 1 × 1 × 2 supercell expansion contains one (Ga,Ge)_2_O_8_ structural unit and resembles the La_3_Ga_3.5_Ge_2.5_O_14.75_ experimental composition, while maintaining the computational cost of the calculations relatively low as opposed to the La_3_Ga_3.5_Ge_2.5_O_14.75_ composition which would require larger supercell expansions.

All calculations were performed using plane-wave DFT^29^ with periodic boundary conditions, as implemented in the CASTEP (version 20.11) code.^49^ On-the-fly generated ultrasoft pseudopotentials^50^ and the Perdew–Burke–Ernzerhof (PBE) exchange-correlation functional^51^ were used. The plane-wave cutoff energy was set to 850 eV, and the Brillouin zone was sampled with either a 2 × 2 × 3 Monkhorst–Pack *k*-point grid for La_3_Ga_5_GeO_14_ or a 2 × 2 × 2 Monkhorst–Pack *k*-point grid for La_3_Ga_4_Ge_2_O_14.5_.^52^ A further increase in the cutoff energy and *k*-point density resulted in changes in energy smaller than 1 meV atom^–1^. The Zeroth-Order Regular Approximation (ZORA) approach^53^ was selected to account for relativistic effects, and the electronic energy was optimized self-consistently with a threshold of 1 × 10^–9^ eV atom^–1^. The atomic coordinates and unit cell parameters of all symmetrically inequivalent configurations were optimized setting the convergence threshold for the maximum energy to 1 × 10^–5^ eV atom^–1^, for the maximum force to 3 × 10^–2^ eV Å^–1^, for the maximum stress to 3 × 10^–2^ GPa and for the maximum displacement to 1 × 10^–3^ Å. During the geometry optimization step, five La_3_Ga_4_Ge_2_O_14.5_ configurations exhibited thermodynamic instability by converging to one of the other structural models already contained in the symmetry-adapted configurational ensemble and were therefore excluded, leaving a total of 490 configurations.

The NMR parameters were computed for the optimized geometries using the GIPAW approach^30,31^ and applying the same parameters as in the geometry optimizations. The absolute shielding tensor **σ** in the crystal frame generated in the calculations can be expressed in terms of the isotropic chemical shielding , the anisotropic chemical shielding , and the asymmetry parameter , where σ_*xx*_, σ_*yy*_, and σ_*zz*_ are the principal components obtained upon diagolization of the symmetric part of **σ** ordered such that |σ_*zz*_ – σ_iso_| ≥ |σ_*xx*_ – σ_iso_ | ≥ |σ_*yy*_ – σ_iso_|. To facilitate comparison between the experimental and computational data, the isotropic and anisotropic chemical shifts, respectively δ_iso,cs_ and δ_aniso,cs_, were determined from the computed σ_iso,cs_ and σ_aniso,cs_ terms using δ_iso,cs_ = σ_ref_ + *m σ*_iso,cs_ and δ_aniso,cs_ = *m σ*_aniso,cs_ with σ_ref_ (^17^O) = 222.02 ppm, *m* (^17^O) = −0.872, σ_ref_ (^71^Ga) = 1442.22 ppm, *m* (^71^Ga) = −0.8206, σ_ref_ (^139^La) = 3460.92 ppm, and *m* (^139^La) = −0.6811 for ^139^La. The σ_ref_ and *m* values were determined using a standard procedure^27^ which also minimizes the systematic errors in the calculations. The calculations yield the traceless electric field gradient tensor ***V*** and its three principal components *V*_*xx*_, *V*_*yy*_, *V*_*zz*_ ordered such that |*V*_*zz*_| ≥ |*V*_*yy*_| ≥ |*V*_*xx*_|. The quadrupolar coupling constant  and quadrupolar asymmetry parameter  are commonly used to express ***V***, where *Q* is the nuclear electric quadrupole moment, *h* is the Planck constant, and *e* is the electron charge. The *C*_Q_ values for ^139^La were calculated using *Q*(^139^La) = (0.206 ± 0.004) × 10^–28^ m^2^,^54−56^ while *C*_Q_ values for the other spins were calculated using the *Q* values implemented in CASTEP 20.11 (see Table S1).

### 2.4 Numerical Simulations

NMR spectra for the different symmetry-adapted configurational ensembles were simulated from the computed NMR parameters (i.e., δ_iso,cs_, reduced anisotropic shift δ_aniso,red,cs_ = δ_*zz*_ – δ_iso,cs_, η, *C*_Q_, and η_Q_) using the SIMPSON package.^57^ MAS NMR spectra were simulated with the gcompute method, while the direct method was used for NMR spectra under static conditions. NMR spectra simulated for each structural model in the symmetry-adapted configurational ensemble were multiplied by a statistical weight and subsequently summed to obtain the total NMR spectrum. The statistical weights take into account the configurational degeneracy of the structural model and, in some cases, its relative energy.

## 3 Results and Discussion

### 3.1 Configurational Disorder

**Figure 2:**
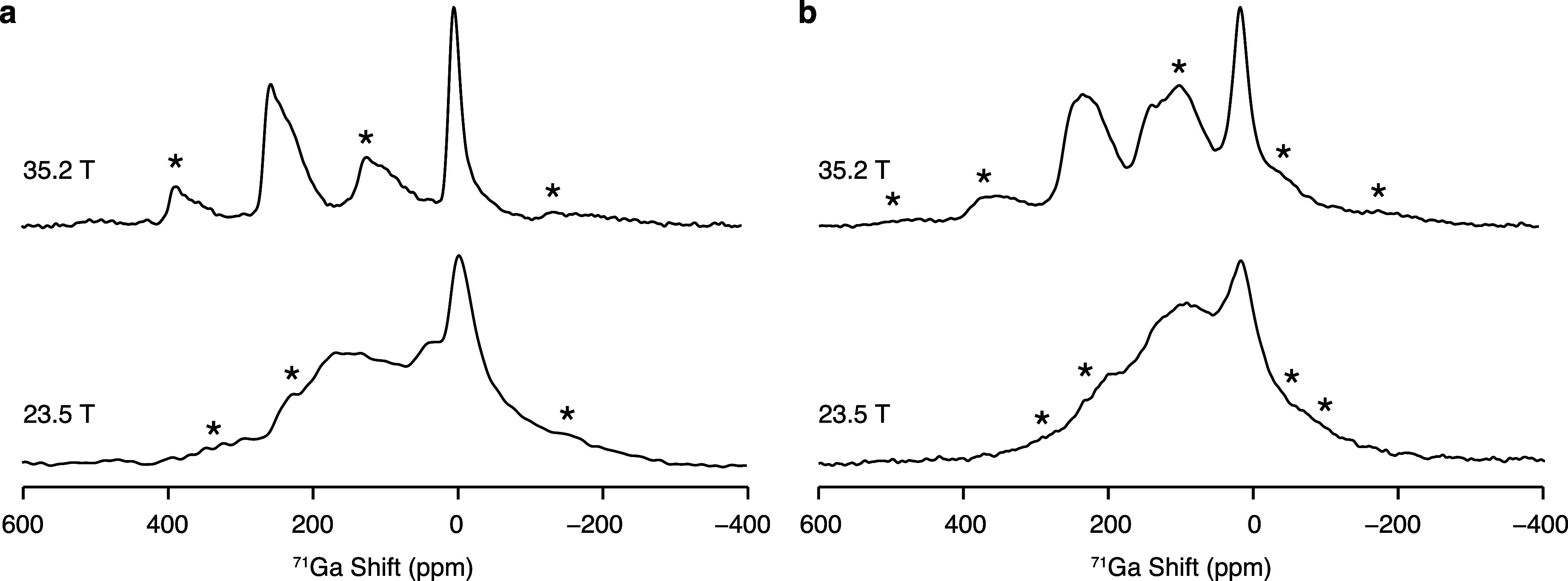
One-dimensional ^71^Ga MAS NMR spectra of (a) La_3_Ga_5_GeO_14_ and (b) La_3_Ga_3.5_Ge_2.5_O_14.75_ recorded at 23.5 T and 35.2 T under ν_r_ = 60 kHz. Data at 35.2 T were recorded with the rotor-synchronized QCPMG sequence processed with coadded echoes. The asterisks (*) denote the spinning sidebands.

Figure 2 shows the ^71^Ga MAS NMR spectra of La_3_Ga_5_GeO_14_ and La_3_Ga_3.5_Ge_2.5_O_14.75_ recorded at 23.5 T and 35.2 T while spinning the samples at ν_r_ = 60 kHz. Ultrahigh field NMR spectroscopy is particularly critical for the detection of half-integer quadrupolar nuclei such as ^71^Ga because strong quadrupolar interactions result in a fourth-rank second-order quadrupolar broadening of the NMR resonances (in Hz) that remains even under MAS but is inversely proportional to the external magnetic field strength *B*_0_. While the ^71^Ga MAS NMR spectra at 23.5 T are dominated by broad, overlapped resonances and show limited gain in resolution with respect to data acquired at 20 T under ν_r_ = 65 kHz,^7^ further increasing the external magnetic field strength by 50% considerably enhances the spectral resolution, thereby enabling the detection of several distinct ^71^Ga resonances at 35.2 T for both La_3_Ga_5_GeO_14_ and La_3_Ga_3.5_Ge_2.5_O_14.75_, as previously observed for the related La_1+*x*_Sr_1–*x*_Ga_3_O_7+0.5*x*_ melilite family of fast oxide ion conductors.^58^

**Figure 3:**
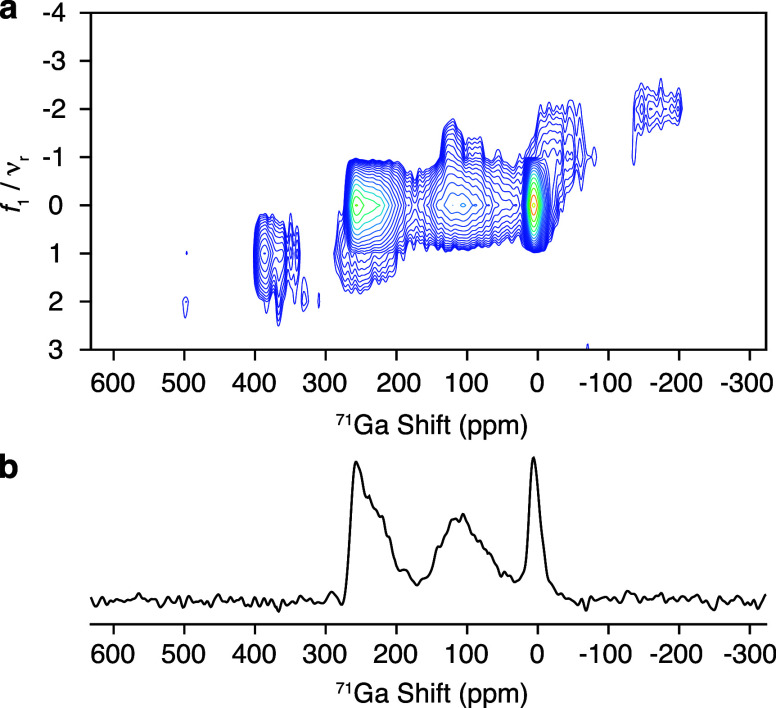
Two-dimensional ^71^Ga QMAT spectrum of La_3_Ga_5_GeO_14_ recorded at 35.2 T under ν_r_ = 60 kHz presented in (a) the Phase-Adjusted Sideband Separation (PASS) representation after shearing the *f*_1_ dimension and (b) the “infinite MAS” representation after shearing (a) along *f*_2_. MATLAB was used to process and shear the data.

One relatively sharp signal at a shift δ of ∼5 ppm and one broader spectral feature in the 190 ppm–270 ppm region are clearly observed for La_3_Ga_5_GeO_14_. Although overshadowed by partial overlap with the spinning sideband manifold, one additional resonance that is unresolved at lower magnetic field strengths is detected at intermediate shifts 50 ppm < δ < 150 ppm. In order to prevent the interference of the spinning sidebands, a two-dimensional ^71^Ga QMAT experiment was performed for La_3_Ga_5_GeO_14_ at 35.2 T (Figure 3).^40^ The QMAT pulse sequence enables complete separation of the spinning sidebands by their order, as shown in Figure 3a. The “infinite MAS” representation of the QMAT data presented in Figure 3b shows the spectrum without spinning sidebands as if acquired under infinitely high MAS rates, and the observed spectral line shape is clear evidence for the presence of three distinct signals in the ^71^Ga MAS NMR data of La_3_Ga_5_GeO_14_. The ^71^Ga MAS NMR spectrum of La_3_Ga_3.5_Ge_2.5_O_14.75_ acquired at 35.2 T, albeit presenting spectral features resembling those observed for La_3_Ga_5_GeO_14_, exhibits broader resonances, reflective of the enhanced structural disorder in the Ge^4+^-doped langasite phase. Furthermore, it is importantly observed that the relative area of the signal at 50 ppm < δ < 150 ppm increases upon Ge^4+^-doping of La_3_Ga_5_GeO_14_ to form La_3_Ga_3.5_Ge_2.5_O_14.75_.

**Figure 4:**
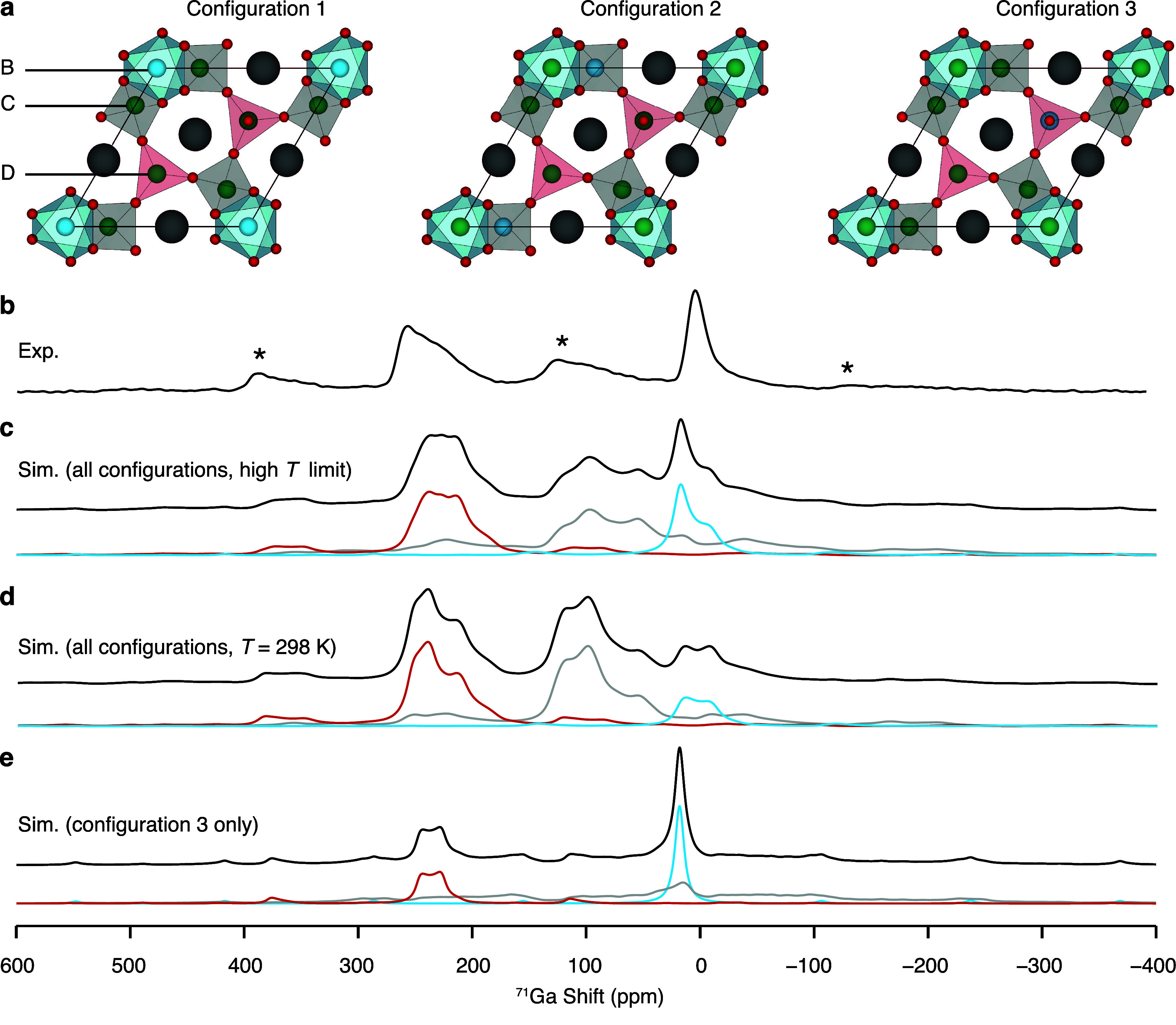
(a) Three symmetrically inequivalent configurations generated with the SOD program from a 1 × 1 × 1 average unit cell of La_3_Ga_5_GeO_14_ with chemical disorder in the B, C and D sites, highlighting three-connected DO_4_ tetrahedra in red, four-connected CO_4_ tetrahedra in gray and BO_6_ octahedra in blue. O, Ga, Ge and La atoms are shown in red, green, blue and gray. (b) Experimental ^71^Ga rotor-synchronized QCPMG spectrum of La_3_Ga_5_GeO_14_ recorded at 35.2 T and processed with coadded echoes. The asterisk symbols (*) denote experimental spinning sidebands. The signal assigned to four-connected CO_4_ tetrahedra overlaps with the spinning sideband at ∼100 ppm. Simulated ^71^Ga MAS NMR spectra of La_3_Ga_5_GeO_14_ (c–d) assuming chemical disorder of the B, C and D sites and (e) constraining Ge^4+^ cations to D sites. The ^71^Ga simulated MAS NMR spectrum was simulated taking into account (c) the configurational degeneracy (high temperature limit) and (d) an additional Boltzmann factor at 298 K. The colored lines indicate the contribution of each site to the simulated spectrum and are color-coded with the polyhedra shown above.

To facilitate the spectral assignment of the ^71^Ga resonances observed for La_3_Ga_5–*x*_Ge_1+*x*_O_14+0.5*x*_, NMR parameters were computed with the GIPAW-DFT method for a symmetry-adapted configurational ensemble generated using the SOD approach.^30−32,59^ Restricting the Ga^3+^/Ge^4+^ mixed site disorder to the D site in La_3_Ga_5_GeO_14_,^11^ only one symmetrically inequivalent configuration is generated starting from a 1 × 1 × 1 average unit cell (Configuration 3 in Figure 4a). The computed ^71^Ga NMR parameters are on the order of those previously predicted for a La_24_Ga_40_Ge_8_O_112_ supercell corresponding to the La_3_Ga_5_GeO_14_ structure, with GaB, GaC, and GaD sites presenting increasing isotropic chemical shifts from ∼18 ppm to ∼253 ppm and GaC exhibiting an extremely large quadrupolar coupling constants of ∼24.6 MHz (Figure S1).^7^ The ^71^Ga MAS NMR spectrum of La_3_Ga_5_GeO_14_ simulated at 35.2 T from the computed NMR parameters and shown in Figure 4e suggests that the relatively sharp resonance at δ of ∼5 ppm and the broader signal in the 190 ppm–270 ppm region correspond to octahedral GaB and tetrahedral GaD sites, respectively, in agreement with the relation between Ga coordination environment and ^71^Ga isotropic chemical shift which indicates that higher coordination numbers yield lower δ_iso,cs_ values.^26−28^ Nevertheless, poor agreement between the experimental and simulated spectra is clearly observed, especially for the signal detected in the 50 ppm–150 ppm spectral region which is assigned to GaC (Figure 4b,e). The particularly large *C*_Q_ constant computed for GaC leads to a severe anisotropic broadening which is considerably greater than that observed experimentally for the GaC signal. Furthermore, the relative area of the GaD signal in the experimental spectrum is larger than that observed for GaB, in contrast with the 1:1 ratio in the computational data obtained for an average unit cell containing equal percentage of GaB and GaD sites.

To address the discrepancies between the experimental and simulated spectra, La_3_Ga_5_GeO_14_ was modeled with additional Ga^3+^/Ge^4+^ mixed site occupancy for the B and C sites, as proposed for the Ge^4+^-doped phase based on neutron powder diffraction.^7^ The three symmetrically inequivalent configurations generated with the SOD approach from the 1 × 1 × 1 average unit cell assuming chemical disorder for B, C, and D sites are shown in Figure 4a, where Configuration 3 corresponds to the structural model previously generated when constraining Ge^4+^ cations to D sites. The NMR parameters were computed for the three symmetrically inequivalent configurations and are shown in Figure S1. While the isotropic chemical shifts are largely unaffected by the Ga^3+^/Ge^4+^ cation distribution, the *C*_Q_ constants computed for GaC sites in Configurations 1 and 2 (15 MHz–20 MHz) are significantly smaller than those obtained for Configuration 3 (∼24.6 MHz). Furthermore, the six-coordinate GaB site exhibits larger *C*_Q_ values in Configuration 2 than in Configuration 3, as expected based on the presence of chemical disorder in the nearby four-coordinate GaC sites for Configuration 2 that results in enhanced structural distortion and electrostatic asymmetry at the octahedral sites.

The simulated ^71^Ga MAS NMR spectrum of La_3_Ga_5_GeO_14_ was obtained as a sum of the spectra computed for each individual configuration weighted by a statistical term which accounts for (i) the degeneracy and (ii) the relative energy of the configurations, the latter expressed by a temperature-dependent Boltzmann factor (). The set of statistical weights were determined both at room temperature, assuming that the configurational ensemble is in thermodynamic equilibrium, and in the high temperature limit , implying an energetically unbiased distribution of the Ga^3+^/Ge^4+^ cations in the disordered material (Table 1). ^71^Ga MAS NMR spectra simulated using statistical weights determined at ambient and infinite temperatures are shown in Figure 4d and Figure 4c, respectively. First, closer agreement between the experimental and computed ^71^Ga MAS NMR spectra of La_3_Ga_5_GeO_14_ is obtained if Ge^4+^ cations are not constrained to the D site (Configuration 3 in Figure 4a), indicating that B, C, and D sites exhibit Ga^3+^/Ge^4+^ mixed site disorder. Second, the predicted spectrum more accurately resembles the experimental data in the high-temperature limit, especially for the GaB resonance owing to the larger statistical weight determined at infinite temperature for the energetically disfavored Configuration 3. This is an indication that the Ga^3+^/Ge^4+^ cation distribution is controlled by the degeneracy of the configurations rather than by their relative energy, implying the occurrence of a kinetically governed cation diffusion process that does not lead to thermodynamic equilibrium.

**Table 1:** Statistical Weights for the Three Symmetrically Inequivalent Configurations Generated from a La_3_Ga_5_GeO_14_ Unit Cell with Chemical Disorder in the B, C, and D Sites^a^

Configuration	*p* (*T* = 298 K)	*p* (*T* → ∞)
1	0.4795	0.1667
2	0.4668	0.5000
3	0.0537	0.3333

a The three configurations are shown in Figure 4a. The weights consider the configurational degeneracy and an additional temperature-dependent Boltzmann factor () determined at 298 K and in the high temperature limit *T* → ∞ (corresponding to ). The temperature dependence of the statistical weights arises from the fact that the configurations possess distinct energies.

The La_3_Ga_4_Ge_2_O_14.5_ composition was chosen to model the Ge^4+^-doped langasite phase because the La_3_Ga_3.5_Ge_2.5_O_14.75_ composition requires a larger supercell expansion that would lead to a prohibitive increase in the computational cost of the calculations. The computed NMR spectrum of La_3_Ga_4_Ge_2_O_14.5_ can be compared with the experimental NMR spectrum of La_3_Ga_3.5_Ge_2.5_O_14.75_ owing to the subtle differences in the ^17^O and ^71^Ga MAS NMR spectra of La_3_Ga_4_Ge_2_O_14.5_ and La_3_Ga_3.5_Ge_2.5_O_14.75_ previously observed.^7^ A symmetry-adapted configurational ensemble consisting of 495 configurations was generated from a 1 × 1 × 2 super cell corresponding to the La_3_Ga_4_Ge_2_O_14.5_ structure (additional details are provided in the Experimental Section). The large amount of structural models arises from the presence of several sites with mixed or partial site occupancy in the average unit cell of the Ge^4+^-doped langasite phase, including Ga^3+^/Ge^4+^ chemical disorder for B, C, D and five-coordinate C^V^ and D^V^ sites and partial site occupancy for the interstitial site O4.^7^

**Figure 5:**
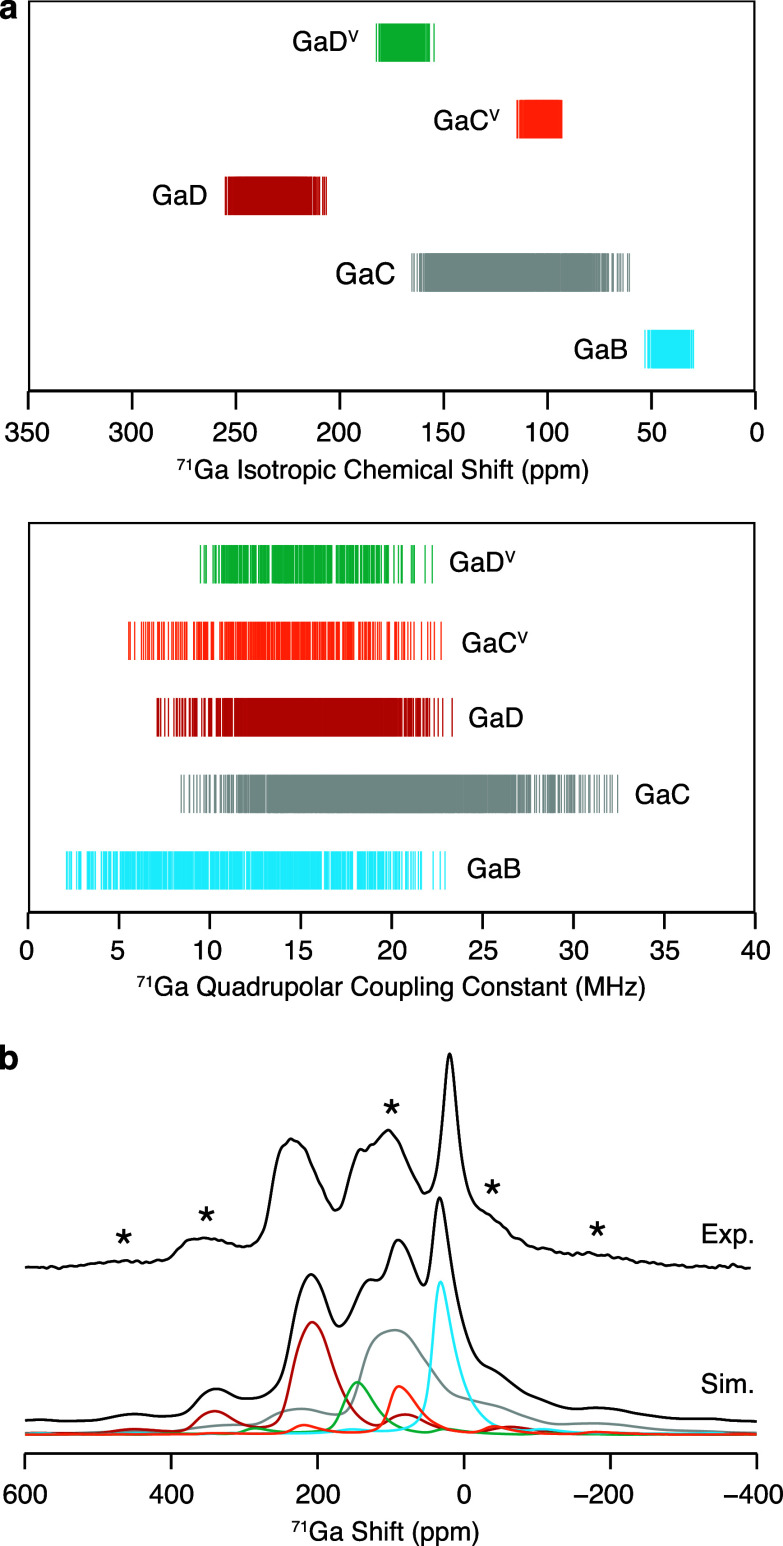
(a) ^71^Ga isotropic chemical shifts and quadrupolar coupling constants computed with the GIPAW approach^30,31^ for a set of symmetrically inequivalent configurations generated with the SOD program^32^ starting from a 1 × 1 × 2 supercell of La_3_Ga_4_Ge_2_O_14.5_. The NMR parameters are grouped according to their site, with six-coordinate GaB, four-coordinate GaC, four-coordinate GaD, five-coordinate GaC^V^, and five-coordinate GaD^V^ sites in blue, gray, red, orange, and green, respectively. (b) Experimental (top) and simulated (bottom) ^71^Ga MAS NMR spectrum of the Ge^4+^-doped langasite phase obtained at 35.2 T and under ν_r_ = 60 kHz (black lines). The simulated spectra are presented in the high temperature limit. The colored lines indicate the contribution of each site to the simulated spectrum and are color-coded with the data presented above. The experimental data were acquired with the QCPMG pulse sequence processed with coadded echoes. The asterisk (*) symbol denotes experimental spinning sidebands.

The ^71^Ga NMR parameters computed for La_3_Ga_4_Ge_2_O_14.5_ are reported in Figure 5a. Although distributed over a wider range due to the presence of enhanced disorder in the Ge^4+^-doped phase, the NMR parameters computed for the four- and six-coordinate Ga sites in La_3_Ga_4_Ge_2_O_14.5_ are of comparable magnitude to those obtained for La_3_Ga_5_GeO_14_. Additional sites are present in La_3_Ga_4_Ge_2_O_14.5_ due to the presence of two edge-sharing, square-based pyramids C^V^O_5_ and D^V^O_5_ which form from the original tetrahedra to accommodate the interstitial oxide ion O4 (Figure 1b).^7^^71^Ga isotropic chemical shifts predicted for GaD^V^ reveal that the incremented coordination number of this site leads to a reduction in the corresponding δ_iso,cs_ value. On the other hand, the isotropic chemical shift computed for five-coordinate GaC^V^ is on the same order of magnitude as δ_iso,cs_ obtained for four-coordinate GaC. While a clear distinction between the ^71^Ga isotropic chemical shifts predicted for GaC^V^ and GaD^V^ is observed, the range of ^71^Ga quadrupolar coupling constants predicted for these sites very significantly overlap (Figure 5a).

The ^71^Ga MAS NMR spectrum of La_3_Ga_4_Ge_2_O_14.5_ was simulated from the NMR parameters in the high temperature limit (Figure 5b), and the computed data are in outstanding agreement with the experimental spectra. Figure 5b indicates that the increase in the relative area of the signal at 50 ppm < δ < 150 ppm experimentally observed upon Ge^4+^-doping originates from the presence of resonances assigned to five-coordinate GaC^V^O_5_ and GaD^V^O_5_ sites that overlap with the GaC signal. These results confirm that the interstitial ions in La_3_Ga_5–*x*_Ge_1+*x*_O_14+0.5*x*_ are hosted in the (Ga,Ge)_2_O_8_ unit consisting of edge-sharing five-coordinate Ga/Ge square pyramidal sites. While structural models with all B sites occupied by Ge^4+^ cations exhibit a dominant Boltzmann factor at ambient temperature due to their favorable relative energy, the six-coordinate ^71^Ga signal is clearly resolved in the experimental spectrum. In the high temperature limit, however, the symmetry-adapted configurational ensemble accurately models the experimental data, implying that the synthesis procedure leads to kinetically controlled Ga^3+^/Ge^4+^ cation distribution (i.e., the Ga^3+^/Ge^4+^ cation distribution does not reach thermodynamic equilibrium when the samples are cooled to ambient conditions after the synthesis procedure), similarly to what was observed for the parent structure.

Due to the sensitivity of this isotope to the local environment, ^73^Ge () NMR spectroscopy is, in principle, ideal to confirm the presence of chemical disorder in the B, C, and D sites for both La_3_Ga_5_GeO_14_ and La_3_Ga_3.5_Ge_2.5_O_14.75_.^60^ Nevertheless, this only NMR active isotope of Ge possesses a large nuclear electric quadrupole moment of (−0.196 ± 0.001) × 10^–28^ m^2^ and suffers from a low natural abundance of 7.76% and a low Larmor frequency of 29.66 MHz at 20 T (resulting in a receptivity *R* (^13^C) of only 1 order of magnitude higher than that of ^17^O at natural abundance, see Table S1). We attempted to record one-dimensional ^73^Ge NMR spectra for La_3_Ga_5_GeO_14_ and La_3_Ga_3.5_Ge_2.5_O_14.75_ at 20 T under static conditions, but the unfavorable NMR properties of ^73^Ge combined with the small Ge content in the samples and the large computed *C*_Q_ values (Figures S2 and S3) prevented the detection of any signal with the available equipment.

**Figure 6:**
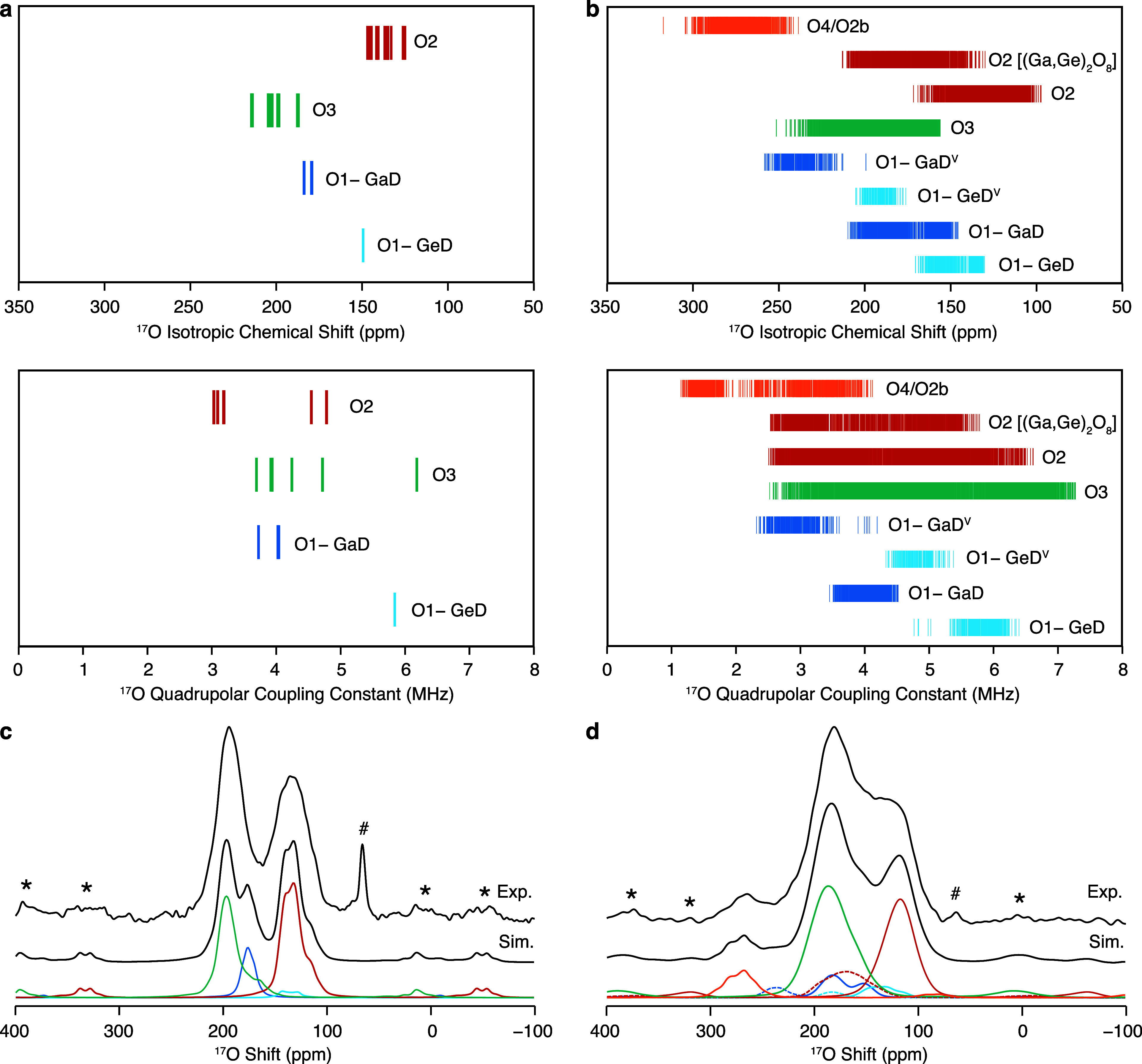
^17^O isotropic chemical shifts and quadrupolar coupling constants computed with the GIPAW approach^30,31^ for a set of symmetrically inequivalent configurations generated with the SOD program^32^ starting from (a) a 1 × 1 × 1 unit cell of La_3_Ga_5_GeO_14_ and (b) a 1 × 1 × 2 supercell of La_3_Ga_4_Ge_2_O_14.5_. The NMR parameters are grouped according to their site as noted in the figure, where O1–GaD/O1–GeD and O1–GaD^V^/O1–GeD^V^ correspond to O1 bound to four- and five-coordinate D sites occupied by Ga/Ge, respectively, and O2 [(Ga,Ge)_2_O_8_] denotes O2 sites in the (Ga,Ge)_2_O_8_ structural unit. Experimental (top) and simulated (bottom) ^17^O MAS NMR spectra of the (c) undoped and (d) Ge^4+^-doped langasite phases obtained at 20 T and under ν_r_ = 22 kHz (black lines).^7^ The simulated spectra are presented in the high temperature limit. The colored lines (color-coded with the NMR parameters) indicate the contribution of each site to the simulated spectrum. Dashed lines are used to identify O1–Ga/GeD^V^ and O2[(Ga,Ge)_2_O_8_] signals. Asterisks (*) denote the experimental spinning sidebands, and the hash symbols (#) mark the sharp signal at approximately 70 ppm assigned to adsorbed H_2_O.

^17^O is another key isotope with the potential of providing compelling insight into the local environment of the langasite structure, as demonstrated by the ^17^O MAS NMR spectra recorded at room temperature in previous work.^7^ The ^17^O NMR parameters predicted for La_3_Ga_5_GeO_14_ and La_3_Ga_4_Ge_2_O_14.5_ using the computational approach described above are presented in Figure 6a and 6b, respectively. The ^17^O isotropic shifts predicted for the different O sites in La_3_Ga_5_GeO_14_ are scattered over distinct ranges. In particular, O2 ions connecting CO_4_ and DO_4_ tetrahedra and O3 ions bridging BO_6_ and CO_4_ polyhedra exhibit the lowest and highest δ_iso,cs_ values, respectively. Interestingly, the NMR parameters calculated for the apical O1 ions bound to D sites are strongly affected by the nature of the cation occupying this site, with O1 bound to GeD exhibiting lower isotropic chemical shifts and higher quadrupolar coupling constants than O1 connected to GaD. The ^17^O MAS NMR spectrum simulated from the NMR parameters in the high temperature limit is in excellent agreement with the experimental spectrum previously acquired at 20 T and under MAS rates of 22 kHz (Figure 6c).^7^ Comparison between the experimental and computational data reveals that the spectral feature detected at approximately 200 ppm corresponds to significantly overlapped O3 and O1–GaD resonances, while the signal at lower shifts arises from O2 and O1–GeD sites.

The NMR parameters computed for La_3_Ga_4_Ge_2_O_14.5_ are of comparable magnitude to those predicted for La_3_Ga_5_GeO_14_, but they are distributed over a wider range, as observed for the ^71^Ga NMR parameters (Figure 6b). The pair of five-coordinate C^V^ and D^V^ sites that forms upon Ge^4+^ doping is connected by one interstitial oxide ion O4 and one largely displaced framework oxide ion O2b which give rise to a strongly deshielded signal with higher δ_iso,cs_ and lower *C*_Q_ values compared to those obtained for the other oxygen sites. Furthermore, it is observed that O1 ions connected to D^V^ sites show higher δ_iso,cs_ and lower *C*_Q_ values than those bound to four-coordinate D sites. Similarly, O2 sites in the (Ga,Ge)_2_O_8_ structural unit present overall higher δ_iso,cs_ and slightly lower *C*_Q_ than those obtained for O2 oxygens bridging two four-connected Ga/Ge sites. Compared to the line shape observed for La_3_Ga_5_Ge^17^O_14_, the presence of the (Ga,Ge)_2_O_8_ structural unit in the Ge^4+^-doped phase leads to broader and more significantly overlapped resonances in the corresponding ^17^O MAS NMR spectrum (Figure 6d). Notably, the computed spectral line shape resembles the experimental spectrum remarkably well, thereby validating (i) the complex defect structure proposed by diffraction methods^7^ and (ii) the accuracy of the symmetry-adapted configurational ensemble in the high temperature limit. Furthermore, the relative area of the computed signals is consistent with that observed in the experimental spectra for both La_3_Ga_5_Ge^17^O_14_ and La_3_Ga_3.5_Ge_2.5_^17^O_14.75_, and this is strong evidence for the attainment of homogeneous ^17^O enrichment.

**Figure 7:**
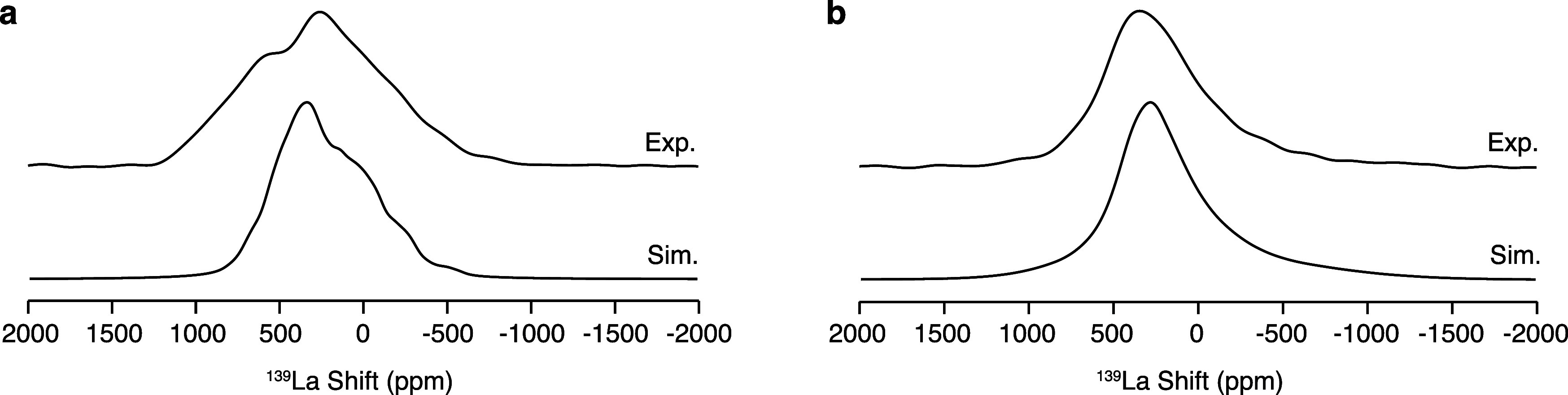
Static ^139^La NMR spectra acquired at 35.2 T with the QCPMG sequence processed with coadded echoes for (a) La_3_Ga_5_GeO_14_ and (b) La_3_Ga_3.5_Ge_2.5_O_14.75_. Spectra simulated in the high temperature limit from the computed NMR parameters are shown below the corresponding experimental data. The NMR parameters computed using the GIPAW approach on a symmetry-adapted configurational ensemble generated from a 1 × 1 × 1 unit cell of La_3_Ga_5_GeO_14_ and a 1 × 1 × 2 supercell of La_3_Ga_4_Ge_2_O_14.5_ are presented in Figure S4.

Further information on the La_3_Ga_5–*x*_Ge_1+*x*_O_14+0.5*x*_ local structure can be provided by solid-state ^139^La NMR spectroscopy. Owing to its large nuclear electric quadrupole moment of (0.206 ± 0.004) × 10^–28^ m^2^, ^139^La is usually subject to strong quadrupolar interactions which result in anisotropically broadened NMR resonances, thereby motivating the use of the highest available fields to achieve enhanced resolution.^54−56^ Static ^139^La NMR spectra of La_3_Ga_5_GeO_14_ and La_3_Ga_3.5_Ge_2.5_O_14.75_ recorded at 35.2 T are shown in Figure 7. One relatively broad signal in the region of the spectrum between −800 and 1200 ppm is observed for both La_3_Ga_5_GeO_14_ and La_3_Ga_3.5_Ge_2.5_O_14.75_, with the signal obtained for La_3_Ga_5_GeO_14_ also exhibiting one shoulder at high frequencies. The absence of spectral features in the ^139^La NMR spectrum for La_3_Ga_3.5_Ge_2.5_O_14.75_ clearly indicates that doping La_3_Ga_5_GeO_14_ with Ge^4+^ leads to enhanced disorder.

Static ^139^La NMR spectra were simulated using the computational approach discussed above (Figure 7). The computed NMR parameters presented in Figure S4 reveal δ_iso_, *C*_Q_, and η_Q_ values scattered over wide ranges for La_3_Ga_4_Ge_2_O_14.5_, while reduced variance is observed for La_3_Ga_5_GeO_14_, reflective of the detected enhanced disorder brought about by Ge^4+^ doping. While the line shapes experimentally observed for La_3_Ga_3.5_Ge_2.5_O_14.75_ are well captured by the computational modeling, close reproduction of the La_3_Ga_5_GeO_14_ experimental spectrum is challenged by contradistinctive features being described by NMR parameters that differ by an amount which is comparable with the accuracy threshold of the calculations (Figure 7). This effect is not observed for La_3_Ga_4_Ge_2_O_14.5_ due to averaging.

^139^La NMR spectra were additionally recorded under fast MAS, resulting in the appearance of a set of spinning sidebands separated by the MAS frequency (ν_r_ = 60 kHz), as shown in Figure S5. “Infinite” MAS spectra were acquired using the QMAT sequence coupled with QCPMG acquisition mode and are presented in Figure S6. While poor signal-to-noise ratio is observed for La_3_Ga_3.5_Ge_2.5_O_14.75_ due to the large magnitude of the corresponding ^139^La quadrupolar coupling constants, the asymmetric line shape detected for La_3_Ga_5_GeO_14_ features a low-frequency tail which is attributed to a Czjzek-like distribution of quadrupolar parameters.^61^ This is further captured in the ^139^La MAS NMR spectra of both La_3_Ga_5_GeO_14_ and La_3_Ga_3.5_Ge_2.5_O_14.75_, which also reveal a distribution of isotropic chemical shifts (Figure S3).

### 3.2 Oxygen Dynamics

**Figure 8:**
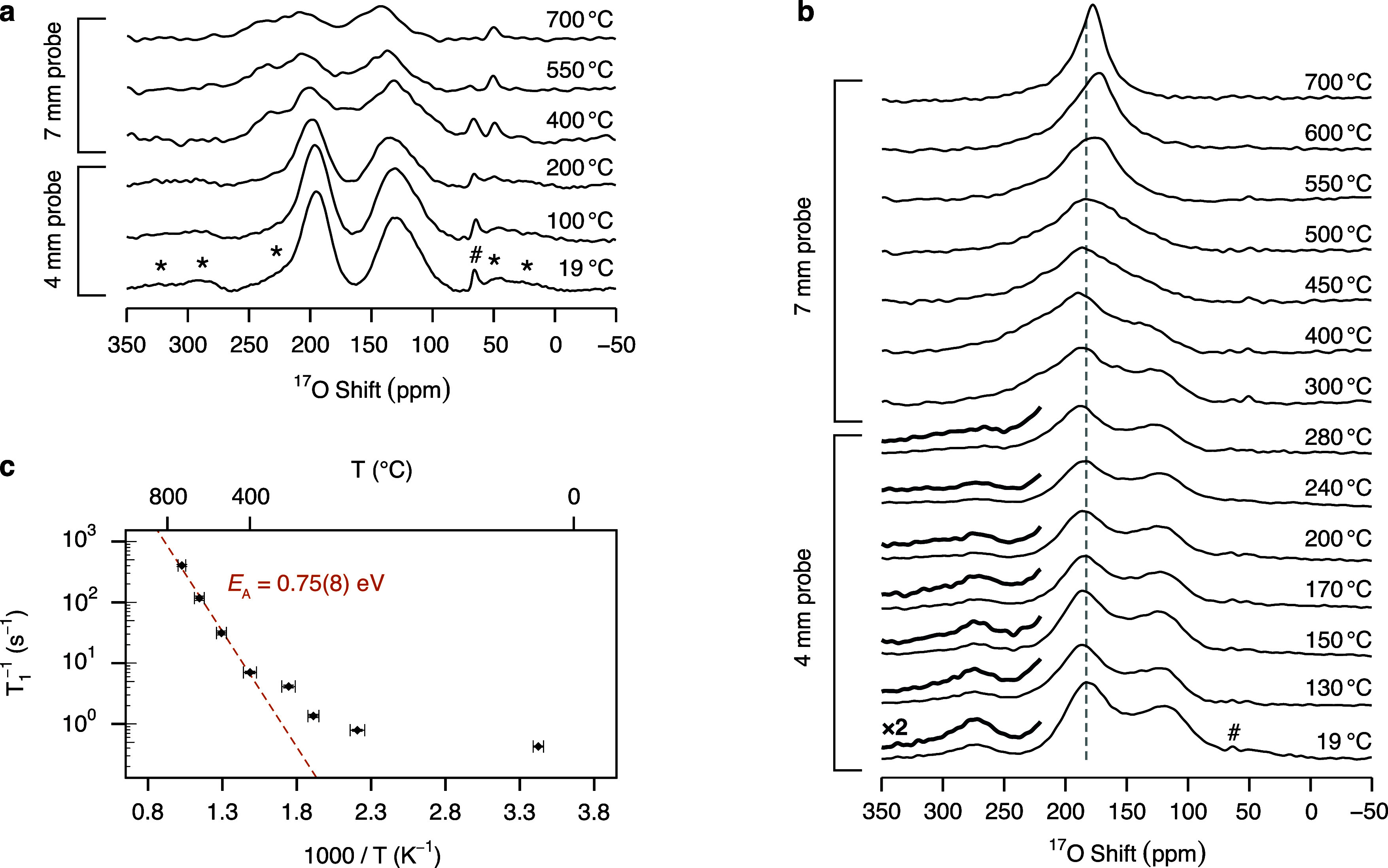
^17^O variable temperature MAS NMR spectra of (a) La_3_Ga_5_Ge^17^O_14_ and (b) La_3_Ga_3.5_Ge_2.5_^17^O_14.75_ recorded at 20 T under a MAS rate ν_r_ of either 10 kHz for La_3_Ga_5_Ge^17^O_14_ and 12.5 kHz La_3_Ga_3.5_Ge_2.5_^17^O_14.75_ (4 mm probe) or 4 kHz (7 mm probe). The asterisk symbols (*) denote spinning sidebands, and the dashed line at 183 ppm in (b) is a guide to the eye. The adsorbed H_2_O signal marked with the hash (#) symbol is observed to move to lower shifts above 400 °C. Magnified views (×2 intensity) of the 220 ppm–350 ppm region containing the O4/O2b signal are shown above the corresponding La_3_Ga_3.5_Ge_2.5_^17^O_14.75_ spectra. In the La_3_Ga_3.5_Ge_2.5_^17^O_14.75_ spectrum at room temperature, the signal centered at ∼183 ppm corresponds to O3 sites, apical O1 sites bound to four- and five-coordinate Ga, apical O1 sites bound to five-coordinate Ge and O2 sites in the (Ga,Ge)_2_O_8_ structural unit, while the resonance at lower shifts is assigned to apical O1 sites bound to five-coordinate Ge and O2 sites. (c) ^17^O spin–lattice relaxation rates of La_3_Ga_3.5_Ge_2.5_^17^O_14.75_ as a function of reciprocal temperature *T* acquired at 20 T under a MAS rate ν_r_ = 4 kHz. The orange dashed line indicates the activation energy *E*_*A*_ for the short-range motion determined from data recorded at *T* > 400 °C.

^17^O VT MAS NMR spectra of La_3_Ga_5_Ge^17^O_14_ (Figure 8a) and La_3_Ga_3.5_Ge_2.5_^17^O_14.75_ (Figure 8b) were recorded to gain insight into differences in the local oxide ion dynamics between the undoped and Ge^4+^-doped langasite phases. ^17^O MAS NMR spectra at *T* < 300 °C were recorded with a 4 mm high temperature probe under ν_r_ = 10 kHz for La_3_Ga_5_Ge^17^O_14_ and ν_r_ = 12.5 kHz for La_3_Ga_3.5_Ge_2.5_^17^O_14.75_, while a 7 mm laser-heated probe spinning at ν_r_ = 4 kHz was employed to acquire data in the 300 °C–700 °C temperature range. Only subtle changes are observed in the ^17^O MAS NMR spectra of La_3_Ga_5_Ge^17^O_14_ as the temperature is increased up to 700 °C. While the center of mass of the spectra is observed to shift to slightly higher chemical shifts at a rate of approximately 0.015 ppm/°C, likely reflecting a small increase in the unit cell parameters and/or a reduction of the quadrupolar coupling constant at high temperatures, the line shape of the resonances is largely not altered by the increase in temperature. The absence of radical changes in the line shape and position of the signals as the temperature is increased is reflective of the poor ionic conductivity known for La_3_Ga_5_GeO_14_.^7^

Striking different behavior is observed for the more highly conductive La_3_Ga_3.5_Ge_2.5_O_14.75_ phase. The ^17^O variable temperature MAS NMR spectra of La_3_Ga_3.5_Ge_2.5_^17^O_14.75_ reveal coalescence of all the ^17^O resonances as the temperature is increased from 20 to 700 °C. The overlapping resonances in the 150 ppm–230 ppm region corresponding to O3 sites, apical O1 sites bound to four- and five-coordinate Ga, apical O1 sites bound to five-coordinate Ge, and O2 sites in the (Ga,Ge)_2_O_8_ structural unit coalesce with the interstitial signal already below 300 °C, while at 450 °C all spectral features coalesce into a single resonance which narrows as the temperature is further increased. This indicates the occurrence of chemical exchange between all oxide ions and supports the involvement of all oxide ions in the conduction mechanism, while also demonstrating that the introduction of interstitial ions in the langasite framework leads to increased ionic motion, in agreement with the enhanced transport properties of La_3_Ga_3.5_Ge_2.5_O_14.75_ compared to La_3_Ga_5_GeO_14_. At the coalescence temperature, the rate τ^–1^ of the detected motion is  where *Δν* is the frequency separation between the resonances in the absence of chemical exchange, yielding values of τ^–1^ up to ∼56 kHz at ∼450 °C.

Comparison of the high temperature ^17^O MAS NMR spectra recorded for the more highly conductive La_1.54_Sr_0.46_Ga_3_^17^O_7.27_ melilite phase and for La_3_Ga_3.5_Ge_2.5_^17^O_14.75_ reveals that the ^17^O resonances in the latter coalesce at higher temperatures.^24^ Considering that the frequency separation of the spectral features in the absence of chemical exchange is comparable for the two compounds, this suggests that the oxide ions are more mobile in the melilite phase, in agreement with the impedance data.^6,7^ This is further supported by the NMR line width of the coalesced signal at 700 °C which is broader for La_3_Ga_3.5_Ge_2.5_O_14.75_ (∼3.2 kHz) than for La_1.54_Sr_0.46_Ga_3_O_7.27_ (∼1.8 kHz). While the small percentage of interstitial defects in La_1.54_Sr_0.46_Ga_3_O_7.27_ hinders the detection of the corresponding signal at high temperatures, the La_3_Ga_3.5_Ge_2.5_^17^O_14.75_ data clearly reveal that the resonance assigned to O4 and O2b ions coalesces with the remaining signals as the temperature is increased, confirming that also the interstitial oxide ions are involved in the detected motional process, as expected.

^17^O spin–lattice relaxation time constants in the laboratory frame of motion *T*_1_ were determined to gain insight into ionic dynamics on the MHz time scale in the langasite phases (fits shown in Figures S7 and S8). The logarithmic *T*_1_^–1^ rates determined for La_3_Ga_5_Ge^17^O_14_ only reveal moderate dependence on the reciprocal temperature and are overall smaller than those obtained for La_3_Ga_3.5_Ge_2.5_^17^O_14.75_, as expected based on the enhanced structural disorder in the Ge^4+^-doped phase (Figure S9). In contrast, the La_3_Ga_3.5_Ge_2.5_^17^O_14.75_ logarithmic *T*_1_^–1^ rates linearly increase with reciprocal temperature above 400 °C (i.e., in the temperature range in which conductivity measurements capture O^2–^ transport),^7^ indicative of the occurrence of thermally activated short-range motion on the MHz time scale (Figure 8c), while below 400 °C the data diverge from a linear trend and show weaker dependence on the temperature. Fitting the linear data to Arrhenius behavior yields an activation energy for the short-range motion equal to (0.75 ± 0.08) eV which, as expected, is lower than the long-range activation energy determined from impedance measurements (∼1.1 eV).^7^ In fact, the short-range oxide ion motion probed with solid-state NMR spectroscopy also captures unsuccessful (i.e., forward and backward) jumps which do not promote the macroscopic anionic diffusion detected in conductivity measurements.^62−64^ The (0.315 ± 0.006) eV activation energy determined for La_1.54_Sr_0.46_Ga_3_O_7.27_ is lower than that determined for La_3_Ga_3.5_Ge_2.5_O_14.75_, further demonstrating the superior ionic transport properties of the melilite phase.^24^

Overall, the high temperature ^17^O MAS NMR experiments confirm that doping La_3_Ga_5_GeO_14_ with Ge^4+^ to form La_3_Ga_3.5_Ge_2.5_O_14.75_ enhances the mobility of the oxide ions by triggering exchange between all oxygen sites. Nevertheless, the oxide ions in La_3_Ga_3.5_Ge_2.5_O_14.75_ are observed to be less mobile than those in La_1.54_Sr_0.46_Ga_3_O_7.27_ melilite. The coalescence of all ^17^O NMR resonances at high temperature importantly indicates the participation of both interstitial and framework oxide ions in the ionic motional process. The ionic diffusion mechanism likely involves the concerted rotation of the polyhedra containing Ga/Ge, leading to randomization of all oxide ions.

## 4 Conclusions

In this work, a combination of experimental and computational multinuclear solid-state NMR approaches are used to investigate the Ga^3+^/Ge^4+^ cation distribution and the ionic diffusion mechanism in the La_3_Ga_5–*x*_Ge_1+*x*_O_14+0.5*x*_ langasite family of oxide ion conductors, the former being particularly challenging to identify using conventional X-ray and neutron diffraction methods. The unique 36 T SCH magnet operating at 35.2 T enables the unambiguous detection of ^71^Ga NMR resonances assigned to Ga sites in four-, five- and sixfold coordination environments, thereby overcoming the resolution limitations encountered at lower magnetic field strengths. The complex spectral line shapes observed in the ^17^O and ^71^Ga experimental MAS NMR spectra are very well reproduced by the NMR parameters computed for a symmetry-adapted configurational ensemble, confirming that excess oxygen in La_3_Ga_3.5_Ge_2.5_O_14.75_ is stabilized by the formation of a (Ga,Ge)_2_O_8_ structural unit, as opposed to the interstitial oxide ions in the La_1.54_Sr_0.46_Ga_3_O_7.27_ melilite which are accommodated in a GaO_5_ structural unit. Comparison of the experimental and simulated NMR spectra reveals that the synthesis procedure results in a kinetically controlled Ga^3+^/Ge^4+^ cation diffusion across the B, C, D, C^V^, and D^V^ sites. This work illustrates that compositional disorder of isoelectronic cations with similar coherent neutron scattering lengths can be unravelled using a combined experimental and computational solid-state NMR approach that does not rely on diffraction-based methodologies.

^17^O MAS NMR spectra at variable temperature up to 700 °C provide insight into the oxygen dynamics. As also concluded for the La_1.54_Sr_0.46_Ga_3_O_7.27_ melilite structure, the coalescence of all ^17^O NMR resonances observed for La_3_Ga_3.5_Ge_2.5_^17^O_14.75_ indicates that (i) the incorporation of interstitial defects in the langasite structure triggers exchange between all oxygen sites and (ii) both framework and interstitial oxide ions play an important role in the conduction mechanism. These results demonstrate the potential of solid-state NMR spectroscopy to capture the relation between short-range structure and anionic conductivity in site-disordered materials.

## Data Availability

Reserach data supporting this work are accessible from the University of Liverpool Research Data Catalogue: https://doi.org/10.17638/datacat.liverpool.ac.uk/2658.

## Supporting Information Available

Additional ^71^Ga, ^73^Ge and ^139^La computed NMR parameters, ^139^La NMR spectra, and ^17^O NMR *T*_1_ relaxation data. (PDF)
