# Supplementary material for: Cation Distribution and Anion Transport in the La3Ga5–xGe1+xO14+0.5x Langasite Structure
Source: J Am Chem Soc. 2024 May 8;146(20):14022–35. doi: 10.1021/jacs.4c02324 (PMC11117410; doi:10.1021/jacs.4c02324)
Supplement: Supplementary file 1 — ja4c02324_si_001.pdf [file ja4c02324_si_001.pdf]

Supplementary Information

Cation Distribution and Anion Transport in the $La_3Ga_{5-x}Ge_{1+x}O_{14+0.5x}$ Langasite Structure

Lucia Corti[1, 2], Ivan Hung[3], Amrit Venkatesh[3], Zhehong Gan[3], John B. Claridge[1, 2], Matthew J. Rosseinsky[1, 2] and Frédéric Blanc[*, 1, 2, 4]

[1]Department of Chemistry, University of Liverpool, Liverpool L69 7ZD, UK

[2]Leverhulme Research Centre for Functional Materials Design, Materials Innovation Factory, University of Liverpool, Liverpool L69 7ZD, UK

[3]National High Magnetic Field Laboratory, Florida State University, Tallahassee FL 32310, USA

[4]Stephenson Institute for Renewable Energy, University of Liverpool, Liverpool L69 7ZF, UK

[*]email: frederic.blanc@liverpool.ac.uk

Table S1: Comparison of the Nuclear Magnetic Resonance (NMR) properties, including spin quantum number I, natural abundance, receptivity with respect to ^{13}C at natural abundance, and nuclear electric quadrupole moment Q of the nuclei probed in this work (i.e., ^{17}O, ^{71}Ga, ^{73}Ge and ^{139}La). The Q values listed in the table were used to derive the corresponding quadrupolar coupling constants.

Nucleus	I	Natural abundance (%)	Receptivity	Q ($\times 10^{-28}$ m^2)
^{17}O	5/2	0.038	6.50×10^{-2}	-0.02558
^{71}Ga	3/2	39.89	$3.35 \times 10^{+2}$	0.107
^{73}Ge	9/2	7.76	6.44×10^{-1}	-0.173
^{139}La	7/2	99.91	$3.56 \times 10^{+2}$	0.206

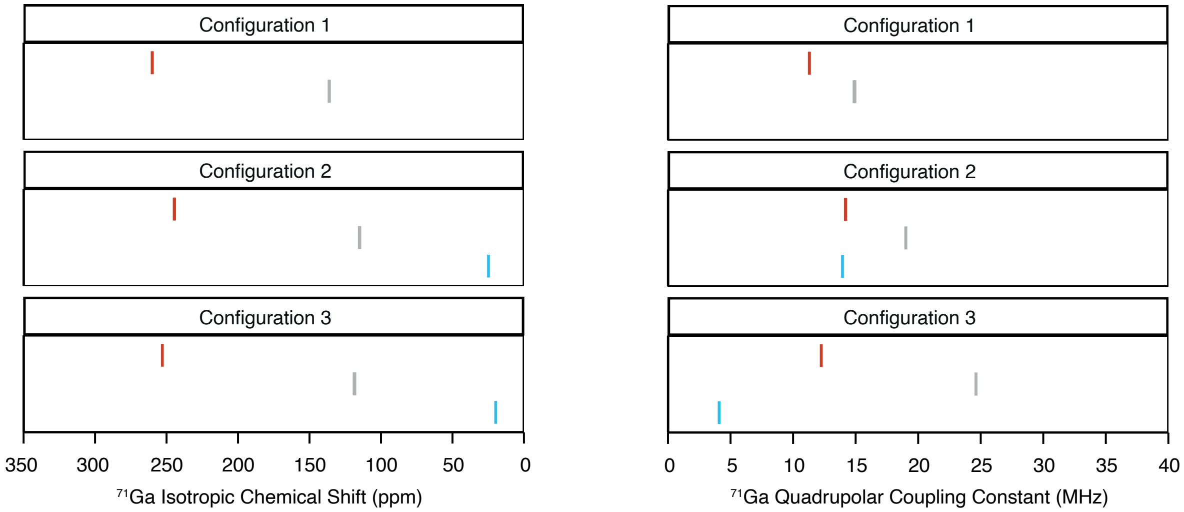

Figure S1: ^{71}Ga isotropic chemical shifts and quadrupolar coupling constants computed with the Gauge Including Projector Augmented Waves (GIPAW) approach[1, 2] for a set of three symmetrically inequivalent configurations generated with the Site Occupancy Disorder (SOD) program[3] starting from a unit cell of $La_3Ga_5GeO_{14}$ with chemical disorder for the B, C and D sites. Data corresponding to three-connected DO_4 tetrahedra, four-connected CO_4 tetrahedra and BO_6 octahedra are shown in red, grey and blue, respectively.

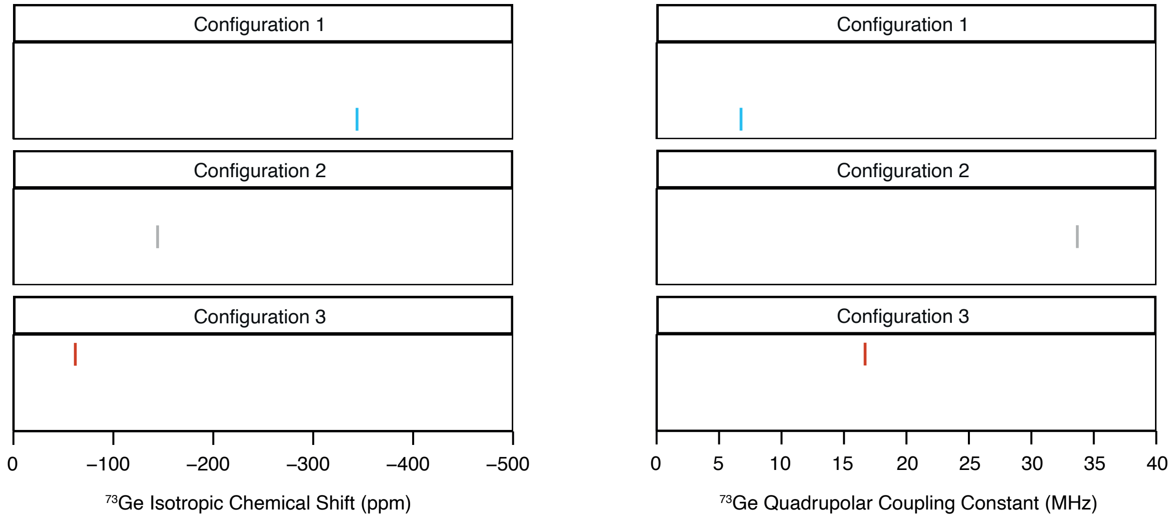

Figure S2: ^{73}Ge isotropic chemical shifts and quadrupolar coupling constants computed with the GIPAW approach[1, 2] for a set of three symmetrically inequivalent configurations generated with the SOD program[3] starting from a unit cell of $La_3Ga_5GeO_{14}$ with chemical disorder for the B, C and D sites. Data corresponding to three-connected DO_4 tetrahedra, four-connected CO_4 tetrahedra and BO_6 octahedra are shown in red, grey and blue, respectively. Approximate values for the isotropic chemical shifts were estimated from the isotropic shieldings using the calculated shielding for quartz GeO_2 as the reference.[4]

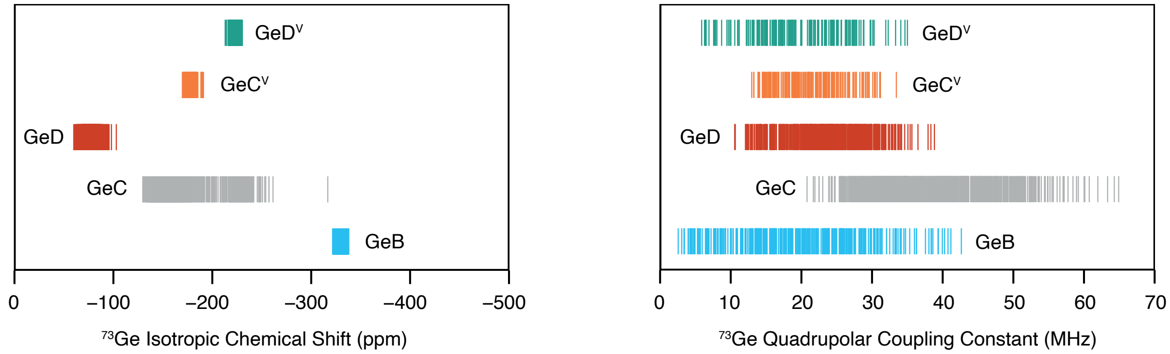

Figure S3: ^{73}Ge isotropic chemical shifts and quadrupolar coupling constants computed with the GIPAW approach[1, 2] for a set of symmetrically inequivalent configurations generated with the SOD program[3] starting from a $1 \times 1 \times 2$ supercell of $La_3Ga_4Ge_2O_{14.5}$. The NMR parameters are grouped according to their site, with six-coordinate GaB, four-coordinate GeC, four-coordinate GeD, five-coordinate GeC^V and five-coordinate GeD^V sites in blue, grey, red, orange and green, respectively. Approximate values for the isotropic chemical shifts were estimated from the isotropic shieldings using the calculated shielding for quartz GeO_2 as the reference.[4]

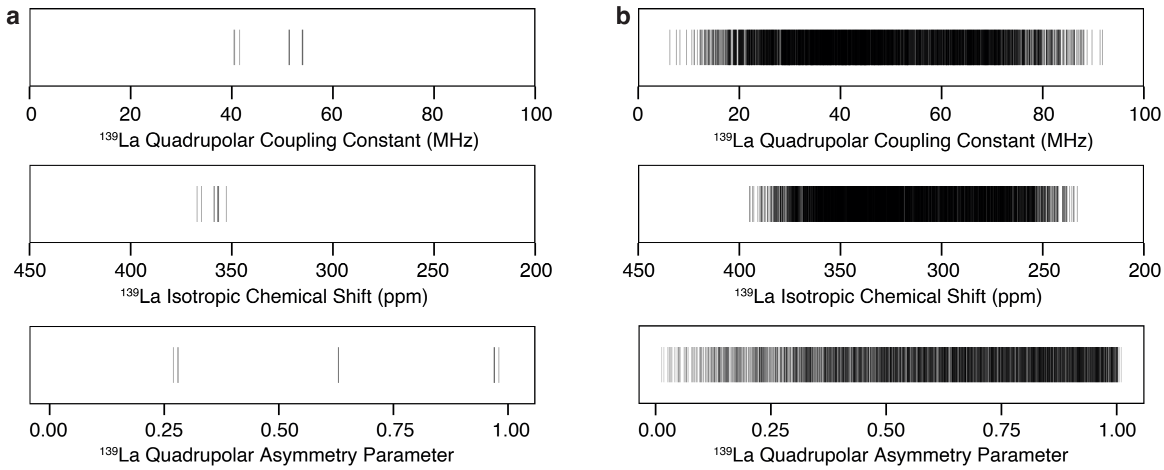

Figure S4: ^{139}La quadrupolar coupling constants, isotropic chemical shifts and quadrupolar asymmetry parameters computed using the GIPAW approach[1, 2] for a symmetry-adapted configurational ensemble generated for (**a**) a $1 \times 1 \times 1$ unit cell of $La_3Ga_5GeO_{14}$ and (**b**) a $1 \times 1 \times 2$ supercell of $La_3Ga_4Ge_2O_{14.5}$. Jitter noise has been added to the ^{139}La quadrupolar asymmetry parameters obtained for $La_3Ga_4Ge_2O_{14.5}$ to avoid exact overlap of data points at regular intervals of 0.01 stemming from the two-decimal rounding precision of the raw data, thereby better illustrating the distribution of the parameters.

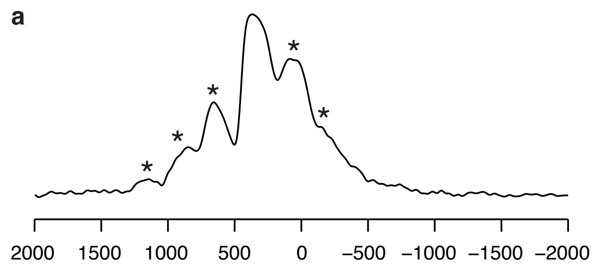

Figure S5: ^{139}La Magic Angle Spinning (MAS) NMR spectra acquired at 35.2 T and under $\nu_r = 60$ kHz with the rotor-synchronized Quadrupolar Carr-Purcell-Meiboom-Gill (QCPMG)[5–8] sequence processed with co-added echoes for (**a**) $La_3Ga_5GeO_{14}$ and (**b**) $La_3Ga_{3.5}Ge_{2.5}O_{14.75}$. The asterisks ($*$) symbol denotes spinning sidebands.

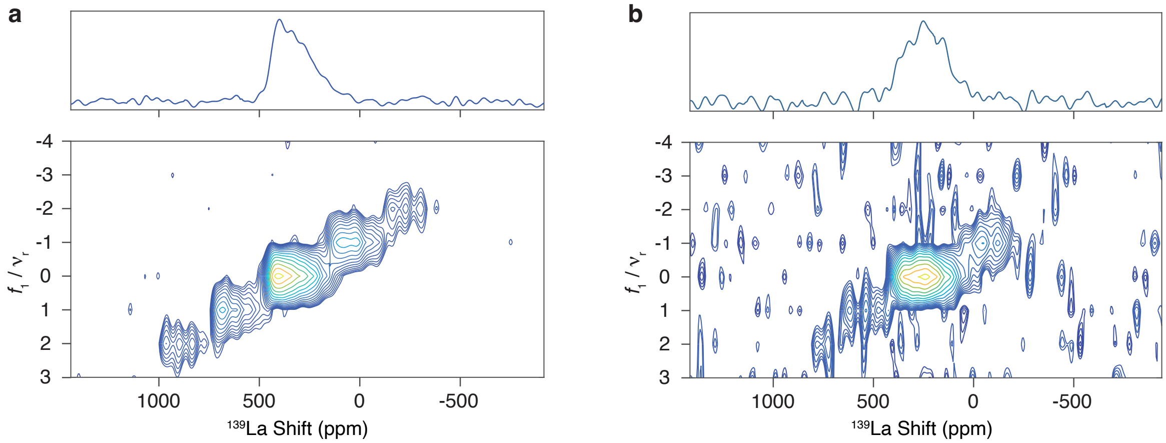

Figure S6: Two-dimensional ^{139}La Quadrupolar Magic-Angle Turning (QMAT)[9] spectra of (**a**) $La_3Ga_5GeO_{14}$ and (**b**) $La_3Ga_{3.5}Ge_{2.5}O_{14.75}$ recorded at 35.2 T under $\nu_r = 60$ kHz presented in the Phase-Adjusted Sideband Separation (PASS) representation after shearing the f_1 dimension (bottom) and in the 'infinite' MAS representation after additional shearing along f_2 (top). `MATLAB` was used to process and shear the data.

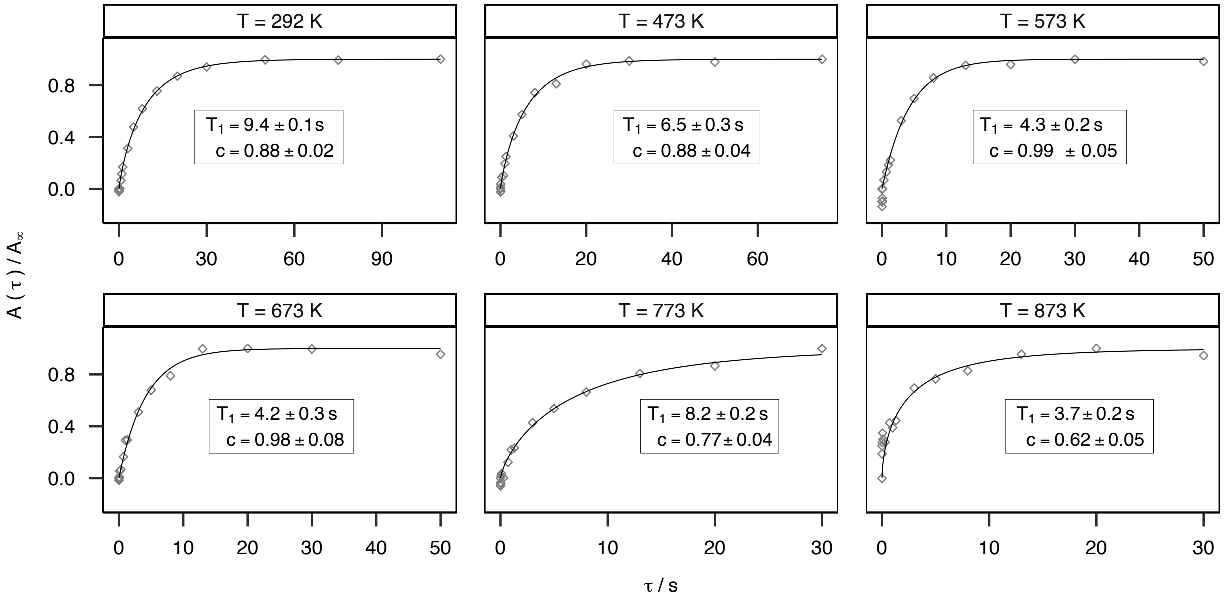

Figure S7: $A(\tau)/A(\infty)$ normalized area of the ^{17}O resonances as a function of delay τ obtained from saturation recovery experiments acquired at 20 T and variable temperature T for $La_3Ga_5Ge^{17}O_{14}$ using a 7 mm laser heated probe under MAS rate $\nu_r = 4$ kHz. Data are fitted to a stretch exponential function. The spin-lattice relaxation time constant T_1 and stretch exponent c are reported in the corresponding plot.

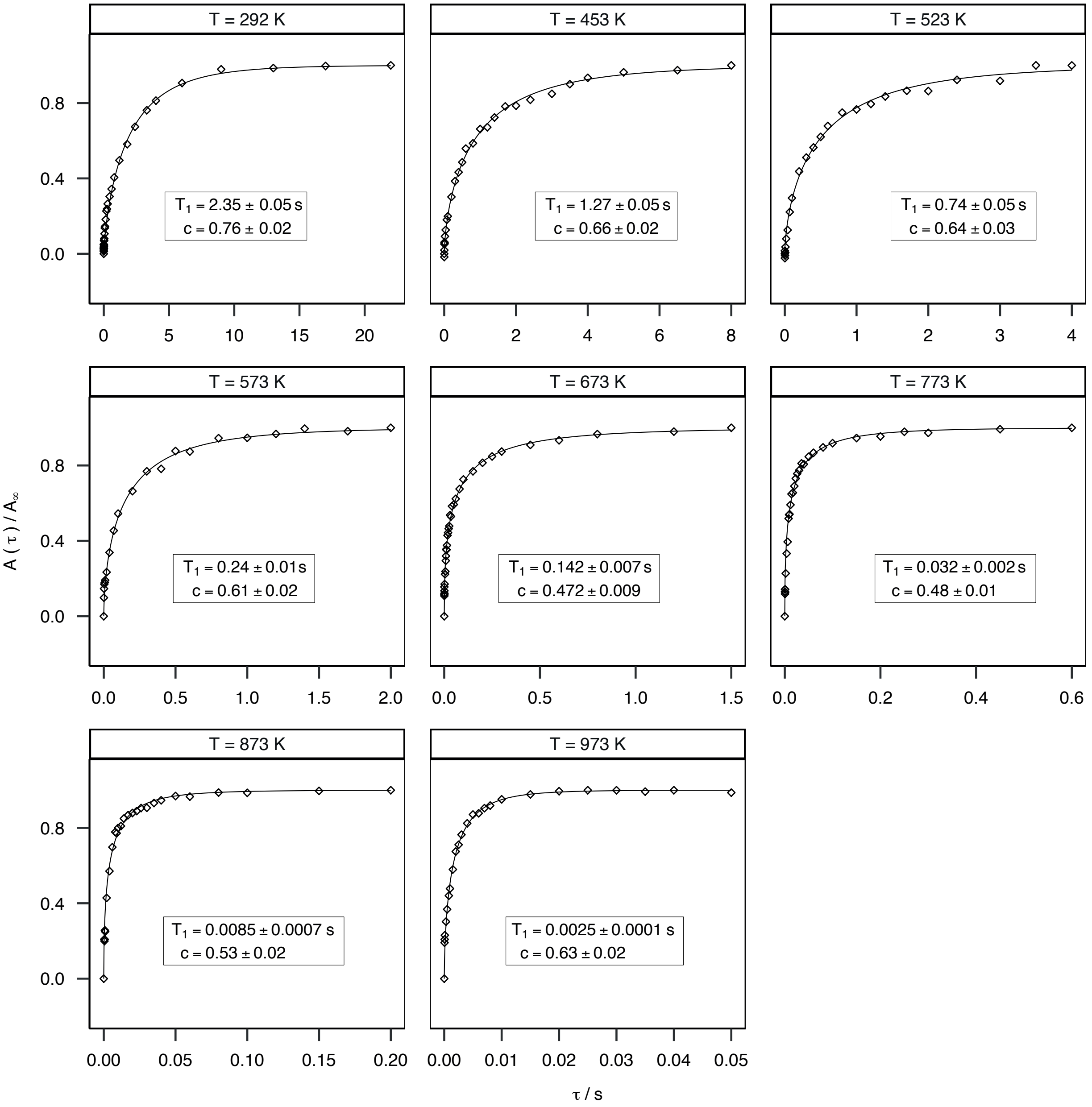

Figure S8: $A(\tau)/A(\infty)$ normalized area of the ^{17}O resonances as a function of delay τ obtained from saturation recovery experiments acquired at 20 T and variable temperature T for $La_3Ga_{3.5}Ge^{17}_{2.5}O_{14.75}$ using a 7 mm laser heated probe under MAS rate $\nu_r = 4$ kHz. Data are fitted to a stretch exponential function. T_1 and c values are reported in the corresponding plot.

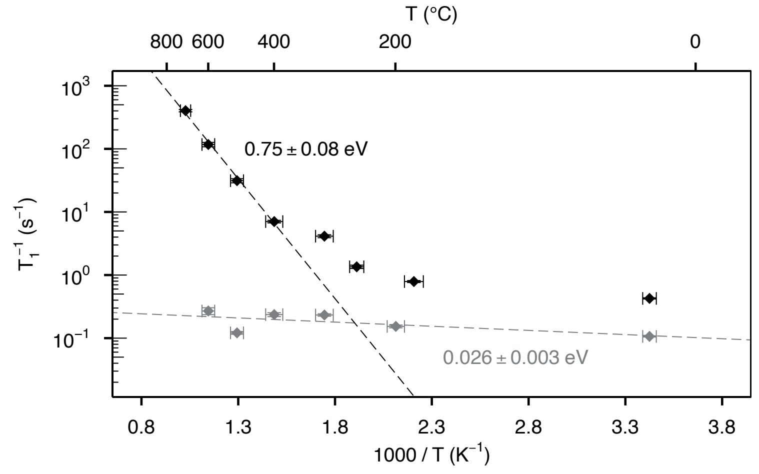

Figure S9: ^{17}O T_1^{-1} rates of $La_3Ga_5Ge^{17}O_{14}$ (gray) and $La_3Ga_{3.5}Ge_{2.5}^{17}O_{14.75}$ (black) as a function of reciprocal temperature $\frac{1000}{T}$ determined from saturation recovery experiments recorded at 20 T and under MAS rate $\nu_r = 4$ kHz. The dashed lines indicates the activation energy E_A for the short-range motion which was determined by fitting the linear $\ln(T_1^{-1})$ vs T^{-1} data to an Arrhenius relationship of the form $\tau^{-1} = \tau_0^{-1} \exp\left(-\frac{E_A}{k_B T}\right)$, where τ^{-1} is the correlation rate of the motion, τ_0^{-1} is the pre-exponential factor, and k_B is the Boltzmann constant.

References

(1) Pickard, C. J.; Mauri, F. All-electron magnetic response with pseudopotentials: NMR chemical shifts. *Phys. Rev. B* **2001**, *63*, 245101, DOI: 10.1103/PhysRevB.63.245101.

(2) Yates, J. R.; Pickard, C. J.; Mauri, F. Calculation of NMR chemical shifts for extended systems using ultrasoft pseudopotentials. *Phys. Rev. B* **2007**, *76*, 024401, DOI: 10.1103/PhysRevB.76.024401.

(3) Grau-Crespo, R.; Hamad Gomez, S.; Catlow, R.; Leeuw, N. Symmetry-adapted configurational modelling of fractional site occupancy in solids. *J. Condens. Matter Phys.* **2007**, *19*, 256201, DOI: 10.1088/0953-8984/19/25/256201.

(4) Kibalchenko, M.; Yates, J. R.; Pasquarello, A. First-principles investigation of the relation between structural and NMR parameters in vitreous GeO_2. *J. Phys.: Condens. Matter* **2010**, *22*, 145501, DOI: 10.1088/0953-8984/22/14/145501.

(5) Carr, H. Y.; Purcell, E. M. Effects of Diffusion on Free Precession in Nuclear Magnetic Resonance Experiments. *Phys. Rev.* **1954**, *94*, 630–638, DOI: 10.1103/PhysRev.94.630.

(6) Meiboom, S.; Gill, D. Modified Spin-Echo Method for Measuring Nuclear Relaxation Times. *Rev. Sci. Instrum.* **1958**, *29*, 688–691, DOI: 10.1063/1.1716296.

(7) Larsen, F. H.; Skibsted, J.; Jakobsen, H. J.; Nielsen, N. C. Solid-State QCPMG NMR of Low-γ Quadrupolar Metal Nuclei in Natural Abundance. *J. Am. Chem. Soc.* **2000**, *122*, 7080–7086, DOI: 10.1021/ja0003526.

(8) Hung, I.; Gan, Z. On the practical aspects of recording wideline QCPMG NMR spectra. *J. Magn. Reson.* **2010**, *204*, 256–265, DOI: 10.1016/j.jmr.2010.03.001.

(9) Hung, I.; Gan, Z. A magic-angle turning NMR experiment for separating spinning sidebands of half-integer quadrupolar nuclei. *Chem. Phys. Lett.* **2010**, *496*, 162–166, DOI: https://doi.org/10.1016/j.cplett.2010.07.016.
